# Association of Alzheimer’s Disease Polygenic Risk Score with Concussion Severity and Recovery Metrics

**DOI:** 10.1007/s40279-024-02150-w

**Published:** 2025-01-16

**Authors:** Kaitlyn M. Dybing, Thomas W. McAllister, Yu-Chien Wu, Brenna C. McDonald, Steven P. Broglio, Jason P. Mihalik, Kevin M. Guskiewicz, Joshua T. Goldman, Jonathan C. Jackson, Andrew J. Saykin, Shannon L. Risacher, Kelly N. H. Nudelman

**Affiliations:** 1 Department of Radiology and Imaging Sciences, Indiana University School of Medicine, Indianapolis, IN USA; 2 Indiana Alzheimer’s Disease Research Center, Indiana University School of Medicine, Indianapolis, IN USA; 3 Stark Neurosciences Research Institute, Indiana University School of Medicine, Indianapolis, IN USA; 4 Department of Neurology, Indiana University School of Medicine, Indianapolis, IN USA; 5 Department of Psychiatry, Indiana University School of Medicine, Indianapolis, IN USA; 6 Department of Medical and Molecular Genetics, Indiana University School of Medicine, Indianapolis, IN USA; 7 Michigan Concussion Center, University of Michigan, Ann Arbor, MI USA; 8 Matthew Gfeller Center, Department of Exercise and Sport Science, University of North Carolina, Chapel Hill, NC USA; 9 Sports Medicine, University of California Los Angeles, Los Angeles, Los Angeles, CA USA; 10 United States Air Force Academy, 2355 Faculty Drive, Suite 1N207, USAFA, CO USA; 11 Utah Valley Orthopedics and Sports Medicine, Provo, UT USA; 12 Utah Valley Orthopedics and Sports Medicine, Saratoga Springs, UT USA; 13 Department of Family Medicine, Uniformed Services University of the Health Sciences, Bethesda, MD USA

## Abstract

**Background:**

Identification of genetic alleles associated with both Alzheimer’s disease (AD) and concussion severity/recovery could help explain the association between concussion and elevated dementia risk. However, there has been little investigation into whether AD risk genes associate with concussion severity/recovery, and the limited findings are mixed.

**Objective:**

We used AD polygenic risk scores (PRS) and *APOE* genotypes to investigate any such associations in the NCAA-DoD Grand Alliance CARE Consortium (CARE) dataset.

**Methods:**

We assessed six concussion outcomes in 931 participants, including two recovery measures (number of days to asymptomatic and to return to play (RTP)) and four severity measures (scores on SAC and BESS, SCAT symptom severity and total number of symptoms). We calculated the PRS using a published score and performed multiple linear regression to assess the relationship of the PRS with outcomes. We also used ANOVAs, *t*-tests, and chi-square tests to examine outcomes by *APOE* genotype.

**Results:**

Higher PRS was associated with longer injury to RTP time in the normal RTP (< 24 days) subgroup (*p* = 0.024). A one standard deviation increase in the PRS resulted in a 9.89 hour increase to RTP time. This result was no longer significant after inclusion of covariates. There were no other consistently significant effects.

**Conclusions:**

Our findings suggest high AD genetic risk is not associated with more severe concussions or poor recovery in young adults. Future studies should attempt to replicate these findings in larger samples with longer follow-up using PRS calculated from diverse populations.

**Supplementary Information:**

The online version contains supplementary material available at 10.1007/s40279-024-02150-w.

## Key Points

**Table:** 

We used AD polygenic risk scores (PRS) and *APOE* genotypes to investigate whether AD genetic risk was associated with concussion severity/recovery in the NCAA-DoD Grand Alliance CARE Consortium dataset. In participants who took less than 24 days to return to play (RTP) after concussion, higher PRS was associated with a longer injury to RTP interval, though this was no longer significant when covariates were included. There were no other consistently significant effects, suggesting that high AD genetic risk is not strongly associated with more severe concussions or poor recovery in young adults.	

## Introduction

Sport-related concussions are a serious public health concern, with an annual occurrence of 1.6–3.8 million in the USA [1–3]. Outside of acute consequences, such as inability to participate in athletic competition and/or academic difficulties [4–7], concussions can be associated with long-term consequences, especially when multiple injuries are incurred [5, 8–13]. Concussion has also been linked to elevated risk for neurodegenerative diseases such as Alzheimer’s disease (AD) [14–16]. However, there have been limited opportunities to examine associations between concussion incurred during the early decades of life and later dementia risk, as longitudinal clinical studies can be costly and challenging [17, 18]. Additionally, dementia research cohorts are often overwhelmingly white and highly educated [19, 20], making it difficult to investigate these effects in diverse populations. The Concussion Assessment, Research and Education (CARE) Consortium was designed to address many of these limitations, and its mission is to expand and improve concussion diagnosis, treatment, and prevention [21]. The CARE dataset is ethnically and racially diverse and includes non-military NCAA collegiate athletes, Military Service Academy students, and Military Service Academy NCAA-student athletes [21].

Previous reports have suggested concussion severity and/or recovery may be influenced by genetic factors. Specifically, the ε4 allele of the apolipoprotein E (*APOE*) gene, also known as *APOE* ε4, is associated with higher likelihood of unfavorable outcome, such as reduced cognitive functioning, after concussion [22–29]. *APOE* plays a critical role in restoration of the blood–brain barrier, regulating inflammation, and clearing waste products after brain injury. Evidence suggests that *APOE* ε4 is less effective in these mechanisms than the neutral *APOE* ε3 allele [30–36], which represents a potential mechanism to explain previously observed associations between *APOE* ε4 and poorer concussion recovery. Importantly, *APOE* ε4 also contributes to elevated risk for AD [37], a progressive neurodegenerative disorder that clinically presents with loss of memory, cognitive function, and behavioral changes. AD is pathologically characterized by extracellular amyloid-β plaques, intracellular neurofibrillary tau tangles, and neurodegeneration [38, 39]. In addition to *APOE* ε4, a myriad of other genetic variants contribute to risk for AD [40]. The overall disease risk of an individual genomic profile can be summarized as a polygenic risk score (PRS), which is a weighted sum of the risk alleles present in an individual [41–43]. PRSs are widely used in both genetic and neurodegenerative disease research [41–43].

The previously described association of *APOE* ε4 with elevated risk for both AD and poor concussion prognosis raises the question of whether additional genetic links may exist. However, previous research has concentrated on *APOE* ε4 [22–28], and other genes are underinvestigated. Therefore, we sought to characterize associations of AD PRS and *APOE* genotype with concussion severity and recovery metrics to assess whether young adult individuals with high genetic risk for AD experience poorer concussion recovery and/or more severe injuries.

## Methods

### Study Sample

This study utilizes data collected from participants in the multisite Concussion Assessment, Research, and Education (CARE) Consortium established by the National Collegiate Athletic Association (NCAA) and US Department of Defense (DoD), protocols of which have been described in previous reports [21]. The study has enrolled over 20,000 participants, though not all incurred an injury while in the study. Upon initiation of this project, there were 1917 recorded concussions in the dataset. Information on these injuries was identified from the “CARE_Injuries_2022_06_28” data file, and a description of the methods used to detect, diagnose, and evaluate these injuries can be found in prior reports [21]. Briefly, NCAA student athletes and Military Service Academy students recruited from participating institutions complete a baseline test battery incorporating demographics, medical history, cognitive performance, and other variables. If at any time point a participant is suspected of having suffered a concussion, evaluation and diagnosis are made by on-site research and medical personnel. Injured participants are then assessed at five timepoints: within 6 h of the injury, again at 24–48 h post-injury, once asymptomatic and cleared to initiate return to play (RTP) protocols, when fully cleared to RTP, and finally approximately 6 months after the injury. Until participants are cleared to return to activity, symptoms are documented daily for up to 14 days, then once weekly thereafter. Tests conducted at each post-injury timepoint include the Sport Concussion Assessment Tool (SCAT) for symptomology and symptom severity, the Standardized Assessment of Concussion (SAC) to assess cognitive performance, the Balance Error Scoring System as a measure of postural stability, and many others. The DoD Human Research Protections Office (HRPO) reviewed and approved all site-level institutional review board (IRB) protocols, and participant consent and site-level IRB approval were also obtained.

Of the 1917 injuries identified at the beginning of this study, 304 did not have associated outcome measures and were removed. Furthermore, for 573 concussions, participants did not have corresponding genetic data and were excluded. Also, only participants’ first injury in the study was considered, thus, 107 repeat injuries were removed due to concern over the introduction of bias on the outcomes and bias due to multiple instances of the same PRS.

We also wanted to remove first- or second-degree relatives from the sample. To do so, we employed the pi-hat identical-by-descent (IBD) estimate from the PLINK software package [44]. Higher pi-hat values represent greater genetic similarity, and we used a threshold of 0.2 to indicate first- or second-degree relatives [44]. In our sample, two pairs of participants had pi-hat > 0.2, and one from each pair was randomly removed. This left 931 non-related injured participants for analysis.

### Participant Stratifications and Subgroup Analyses

**Fig. 1:**
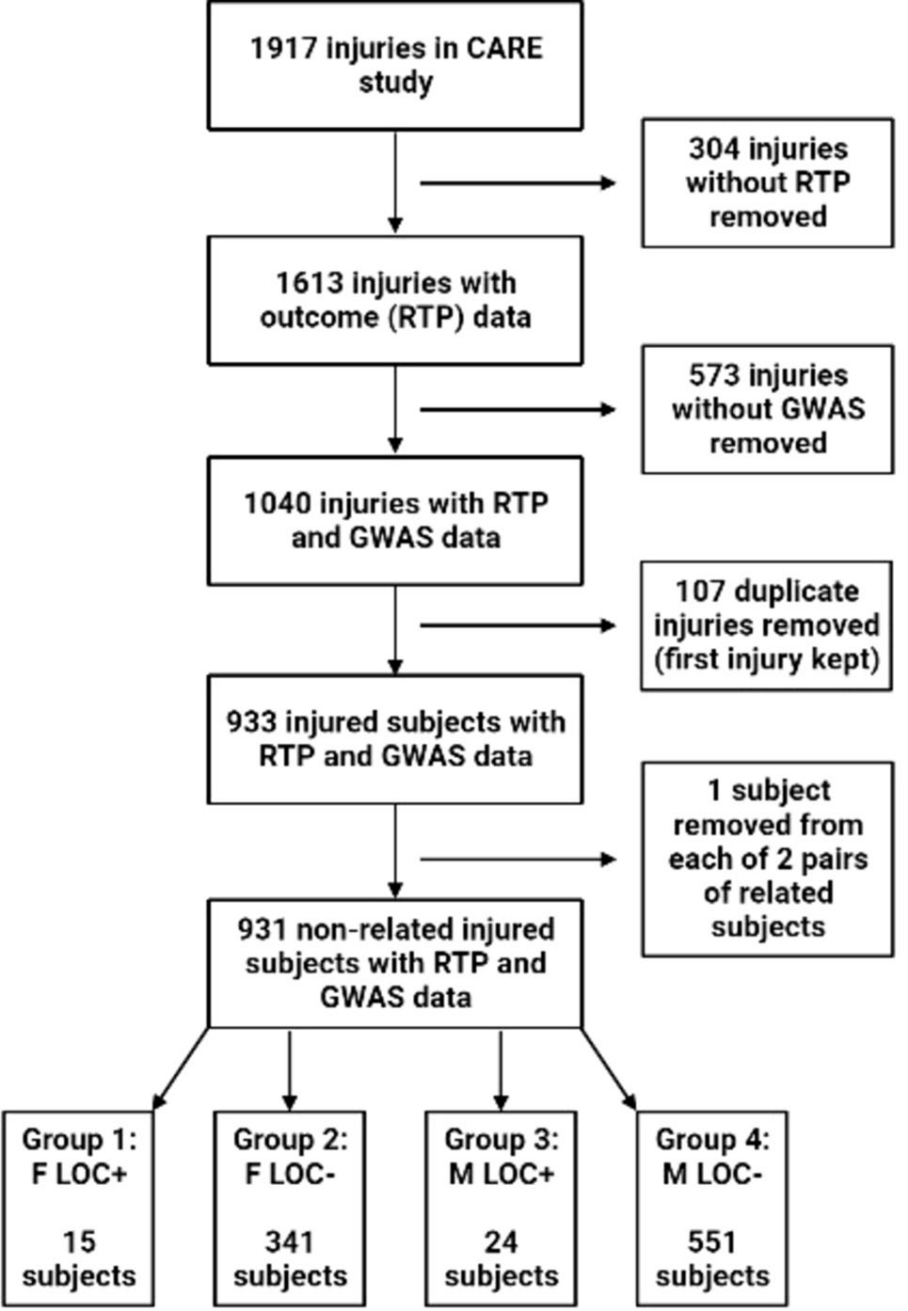
A consort diagram detailing the cohort of Concussion Assessment, Research and Education (CARE) Consortium participants used in this study (created with BioRender.com). *RTP* return to play, *GWAS* genome-wide association study, *F LOC+* female participants with loss of consciousness (LOC), *F LOC−* female participants without LOC, *M LOC+* male participants with LOC, *M LOC−* male participants without LOC

Some reports have suggested there may be sex differences in concussion severity and recovery [45–47]. Similarly, studies have suggested that loss of consciousness (LOC) may be used as a proxy for more severe injury, which can impact recovery trajectories [48, 49]. Therefore, when significant associations were observed between PRS and outcomes in the full participant sample, we further examined the relationships for differential effects based on sex or LOC by stratifying participants on these variables. Participants were subdivided into four groups: females with loss of consciousness (F LOC+), females without loss of consciousness (F LOC−), males with loss of consciousness (M LOC+), and males without loss of consciousness (M LOC−) (Fig. 1). The lack of signal in most of our analyses resulted in this subgrouping being utilized for analysis only once, though we also utilized this subgrouping in our baseline assessments of demographic and neuropsychological variables to establish whether sex- and/or severity-specific effects were visible immediately after injury. A consort diagram of the participant selection process is available as Fig. 1.

We also elected to analyze outcomes with participants stratified by genetically determined ancestry, principally to assess the transferability of the European-derived PRS to non-European CARE participants. To determine genetic ancestry, genome-wide association study (GWAS) data from the CARE participants was compared to 1000 Genomes Reference data using plink multidimensional scaling, which clusters participants into ancestral groups including European (EUR), East Asian (EAS), African (AFR), South Asian (SAS), and admixed Americans (AMR) [50, 51]. The majority of our sample was EUR (731 participants), and the second-largest ancestry group was AFR (135 participants).

Finally, we also elected to perform subgroup analyses based on whether an individual came into the study as a military or civilian participant. This stratification was exploratory and intended to ascertain whether the selected PRS performed differently in certain groups of participants. If differential associations between PRS and outcomes were observed in the military or civilian subgroups, this would prompt further consideration of whether the chosen PRS was appropriate for this sample.

### Determining Polygenic Risk Score and APOE Genotype

To calculate AD PRS, we selected a published score [52] comprising 39 single-nucleotide polymorphisms (SNPs) and used it to calculate PRS in our participants. However, this score was derived entirely from individuals of European ancestry, and the CARE study sample is more diverse. While searching for a PRS to apply in this study, we were unable to identify a PRS that matched the demographics of the CARE study. We, therefore, selected this score principally because of the large sample size (over 400,000) from which it was derived [52], with the caveat that subgroup analyses would be performed in the CARE sample based on genetically derived ancestry to assess the transferability of the PRS to diverse participants in CARE.

Once calculated, PRS were transformed into *z*-scores. The CARE data was missing two alleles from the original PRS: rs2732703 (a duplicated variant with different weights for *APOE* ε4 carriers and noncarriers) and rs616338. We, therefore, calculated the PRS from 37 SNPs in our participants. There were several instances where a participant did not have available data for one or more SNP(s). There are two analytical strategies for addressing this: the missing SNP can be ignored and the PRS calculated without it, or the value of the missing SNP can be substituted with the mean from all other participants on that SNP. To compare these methods, we performed a sensitivity analysis and did not identify significant differences between these strategies (Supplementary Fig. 1a–d). We, therefore, chose to use the ignored missing values strategy for all further analyses. Supplementary Table 2 lists how many participants were missing data for each SNP. The average number of missing SNPs per participant in our study sample was 2.48.

We also wanted to investigate recovery/severity outcomes as a function of *APOE* genotype. As we had limited power, we split participants into *APOE* ε3/ε3, ε2 carriers, and ε4 carriers rather than considering every possible *APOE* genotype. Due to limited power, we chose not to further stratify participants. Of the 931 participants, 10 did not have available *APOE* genotypes and 21 were *APOE* ε2/ε4. The ε2/ε4 participants were excluded given the small number and potential for confounding if included in either the ε2 or ε4 carrier group. Therefore, 900 total participants were included.

### Selection of Outcome Metrics

There is a significant amount of data concerning both concussion severity and recovery generated through the CARE Consortium [21]; we examined six key metrics. These were the injury to asymptomatic interval, the injury to RTP interval, SCAT symptom severity and total symptom scores, and total scores on the SAC and BESS.

First, the injury to return to play (RTP) interval, also called days to RTP or simply RTP, represents the length of time an individual takes to be medically cleared for full participation in sports/physical activity after a concussion [53, 54]. This is thought to typically occur within one month of injury [5, 55–60], but may vary depending on factors such as age [61, 62] and sex [45, 47, 63, 64]. Athletes enrolled in the CARE Consortium initiated the RTP protocol at the discretion of staff and were not necessarily entirely asymptomatic when the protocol was initiated [65]. Though the international consensus group on concussion in sport defined normal recovery to be < 28 days [6], a previous report from the CARE Consortium suggested 80% of CARE participants RTP within 24 days [54]. Therefore, we used < 24 days as the definition of normal RTP in this study. We also considered the injury to asymptomatic interval, which tracks how long a participant remains symptomatic after an injury [54, 60]. In previous studies by the CARE team, 80% of participants were found to be asymptomatic by day 14 after concussion [54].

The Sport Concussion Assessment Tool (5th edition) (SCAT5) is one of the most commonly used tests to evaluate concussion. The SCAT5 incorporates elements such as reading, memory, balance, and gait [66]. Individuals self-report 22 concussion symptoms on a 7-point Likert scale, ranging from 0 (none) to 6 (severe) [66, 67]. This generates a score out of 22 reflecting the total number of symptoms experienced by the individual (SCAT5 total symptoms score) and a score out of 132 indicating the severity of the present symptoms (SCAT5 symptom severity score) [67]. The SCAT5 incorporates the BESS and SAC (described below); from this test we utilized only the SCAT5 symptom severity and total symptoms scores.

Another validated and widely used test for assessing concussions is the Balance Error Scoring System (BESS), which consists of 3 stances performed on both a firm and a foam surface with the eyes closed [68]. Participants are scored from 0 to 30 based on the number of errors made within 20-second trials, with higher scores indicating greater postural instability [68]. Errors include opening the eyes or falling out of the stance, among others [68]. The BESS can identify balance deficits/postural instability in concussed individuals [69–71].

Finally, the Standardized Assessment of Concussion (SAC) is a brief screening tool to assess cognition in suspected concussion through four domains (orientation, immediate memory, concentration, and delayed recall) [72]. Participants receive a total score out of 30, where lower scores indicate poorer performance [72].

### Statistical Analyses

Statistical tests were performed using RStudio 2023.12.0 and SPSS 29.0.1.0. A *p* value < 0.05 was considered significant for all comparisons. Demographic comparisons were performed in SPSS using chi-square tests or one-way analysis of variance (ANOVAs) with Tukey post hoc tests. Chi-square tests were used to determine the expected number of participants in each category relative to the actual number of participants in each category. If the homogeneity of variance assumption was violated (indicated by a significant Levene statistic), the Welch test was used in place of ANOVA, and post-hoc tests were performed using the Games–Howell method. When testing the association of AD PRS with selected outcomes, we performed linear regressions using the lm() function in R. When testing differences in selected outcomes by *APOE* genotype, *t* tests were performed using the t_test() function in R or one-way ANOVAs using SPSS. We also replicated our analyses of the outcomes by PRS and *APOE* genotype in the full sample with covariates of genetic ancestry, sex, LOC status, and participant origin (military versus civilian).

## Results

### Demographic and Neuropsychological Variables

Participant characteristics are summarized in Table 1 and Supplementary Table 1. We analyzed participants’ baseline demographics and neuropsychological characteristics using the first available test values obtained within 24–48 h following the injury (Table 1). F LOC− participants were younger (19.849 years) than M LOC− participants (20.196 years) (mean difference − 0.347 years, *p* < 0.001, 95% confidence interval [CI] − 0.572 to − 0.121). Additionally, F LOC− participants had higher SCAT total symptoms scores (10.276) than M LOC+ participants (5.333) (mean difference 4.942, *p* = 0.028, *η*^2^ = 0.018, 95% CI 0.385–9.500). Furthermore, F LOC− participants took longer to become asymptomatic (12.521 days) than M LOC− participants (9.541 days) (mean difference 2.980, *p* = 0.014, 95% CI 0.431–5.530).

**Table 1:** Baseline demographic and neuropsychological characteristics (at first available post-injury assessment) of included participants

	F LOC+	F LOC−	M LOC+	M LOC−	*N* (%)	Levene statistic (*p*)	ANOVA *F*/Welch statistic	*p* value
Number of participants	15	341	24	551	931	–	–	–
Military participants (%)	0 (0)	70 (20.528)	12 (50)	176 (31.942)	258 (27.712)	–	–	–
Civilian participants (%)	15 (100)	271 (79.472)	12 (50)	375 (68.058)	673 (72.288)	–	–	–
Avg. PRS *z*-score (SD)	− 0.073 (0.542)	− 0.004 (1.026)	0.045 (1.112)	0.003 (0.991)	931	2.006 (0.112)	0.046	0.987
*APOE* genotype								
ε3/ε3	9	206	13	320	548 (58.861)	*χ*^2^ (12, *N* = 931) = 17.950, *p* = 0.117		
ε2 carrier	2	44	2	61	109 (11.708)			
ε4 carrier	4	89	8	142	243 (26.101)			
ε2/ε4 (excluded)	0	1	0	20	21 (2.256)			
Missing	0	1	1	8	10 (1.074)			
Self-reported race/ethnicity (%)								
Non-Hispanic white	10 (66.667)	213 (62.463)	17 (70.833)	320 (58.076)	560 (60.150)	*χ*^2^ (21, *N* = 931) = 36.064, ***p* = 0.022** F LOC− Black/African American (expected 49) M LOC− Black/African American (expected 79)		
Black/African American	2 (13.333)	23 (6.745)	4 (16.667)	104 (18.874)	133 (14.286)			
Hispanic white	1 (6.667)	14 (4.106)	0	18 (3.267)	33 (3.545)			
Asian	0 (0)	8 (2.346)	0	9 (1.633)	17 (1.826)			
Multiple races	2 (13.333)	34 (9.971)	0	39 (7.078)	75 (8.056)			
Hawaiian/Pacific Islander	0	3 (0.880)	0	2 (0.363)	5 (0.537)			
Native American/Indian/Alaskan	0	0	0	1 (0.181)	1 (0.107)			
Skipped	0	46 (13.490)	3 (12.500)	58 (10.526)	107 (11.493)			
Genetic ancestry								
European/EUR	12	280	20	419	731 (78.518)	*χ*^2^ (3, *N* = 866) = 16.067, ***p* = 0.001** F LOC− (expected 261 EUR, 48 AFR) M LOC− (expected 440 EUR, 81 AFR)		
African/AFR	1	29	3	102	135 (14.501)			
Outcomes								
Average age at injury (SD)	20.351 (1.623)	19.849 (1.117)	19.837 (1.024)	20.196 (1.354)	931	4.866** (0.002)**	6.026^a^	**0.002**^a^ (F LOC− versus M LOC-; ***p* < 0.001**)
Average days to RTP (SD)	17.500 (10.455)	23.627 (23.483)	20.038 (21.632)	20.270 (26.109)	931	1.478 (0.219)	1.433	0.232
Average days to asymptomatic (SD)	9.740 (5.264)	12.521 (16.502)	10.365 (20.362)	9.541 (7.812)	857	**7.689 (< 0.001)**	2.938^a^	**0.045**^a^ (F LOC− versus M LOC− ; ***p* = *0.014***)
Average SCAT # of symptoms (SD) *(n)*	9.444 (4.475) *(9)*	10.276 (5.936) *(225)*	5.333 (6.527) *(12)*	9.609 (6.013) *(225)*	471	0.672 (0.569)	2.805	**0.039** (F LOC− versus M LOC+ ; ***p* = 0.028**)
Average SCAT symptom severity score (SD) *(n)*	19.333 (12.708) *(9)*	22.907 (20.238) *(225)*	10.667 (17.201) *(12)*	20.609 (19.419) *(225)*	471	1.201 (0.309)	1.784	0.149
Average BESS total score (SD) *(n)*	12.267 (5.561) *(15)*	14.331 (7.715) *(323)*	13.957 (7.601) *(23)*	14.767 (7.451) *(527)*	888	0.682 (0.563)	0.744	0.526
Average SAC total score (SD) *(n)*	25.067 (6.734) *(15)*	26.952 (2.080) *(336)*	26.708 (2.156) *(24)*	26.762 (2.405) *(543)*	918	12.628 **(< 0.001)**	0.866^a^	0.466

Bolded values indicate that the results met statistical significance at *p* < 0.05

*F LOC+* female participants with loss of consciousness (LOC), *F LOC−*, female participants without LOC, *M LOC+* male participants with LOC, *M LOC−* male participants without LOC, *n* number of participants, *AD* Alzheimer’s disease, *PRS* polygenic risk score, *SD* standard deviation, *RTP* return to play, *SCAT* Sport Concussion Assessment Tool, *BESS* balance error scoring system, *SAC* standardized assessment of concussion

^a^Welch statistic and Welch test *p* value reported due to significant Levene statistic

The chi-square test of self-reported ancestry was significant (*χ*^2^ (21, *N* = 931) = 36.064, *p* = 0.022, Cramer’s *V* = 0.114). There were fewer Black/African American F LOC− participants and more Black/African American M LOC− participants than anticipated. The chi-square test of genetic ancestry was also significant (*χ*^2^ (3, *N* = 866) = 16.067, *p* = 0.001, Cramer’s *V* = 0.136). In the F LOC− group, there were more European participants but fewer African participants than anticipated. Conversely, in the M LOC− group, there were fewer European participants but more African participants than anticipated. The chi-square test of *APOE* genotype was not significant (*χ*^2^ (12, *N* = 931) = 17.950, *p* = 0.117), and there were no additional differences in the outcomes.

### Concussion Recovery (Injury to RTP and Injury to Asymptomatic Intervals) as a Function of PRS

**Fig. 2:**
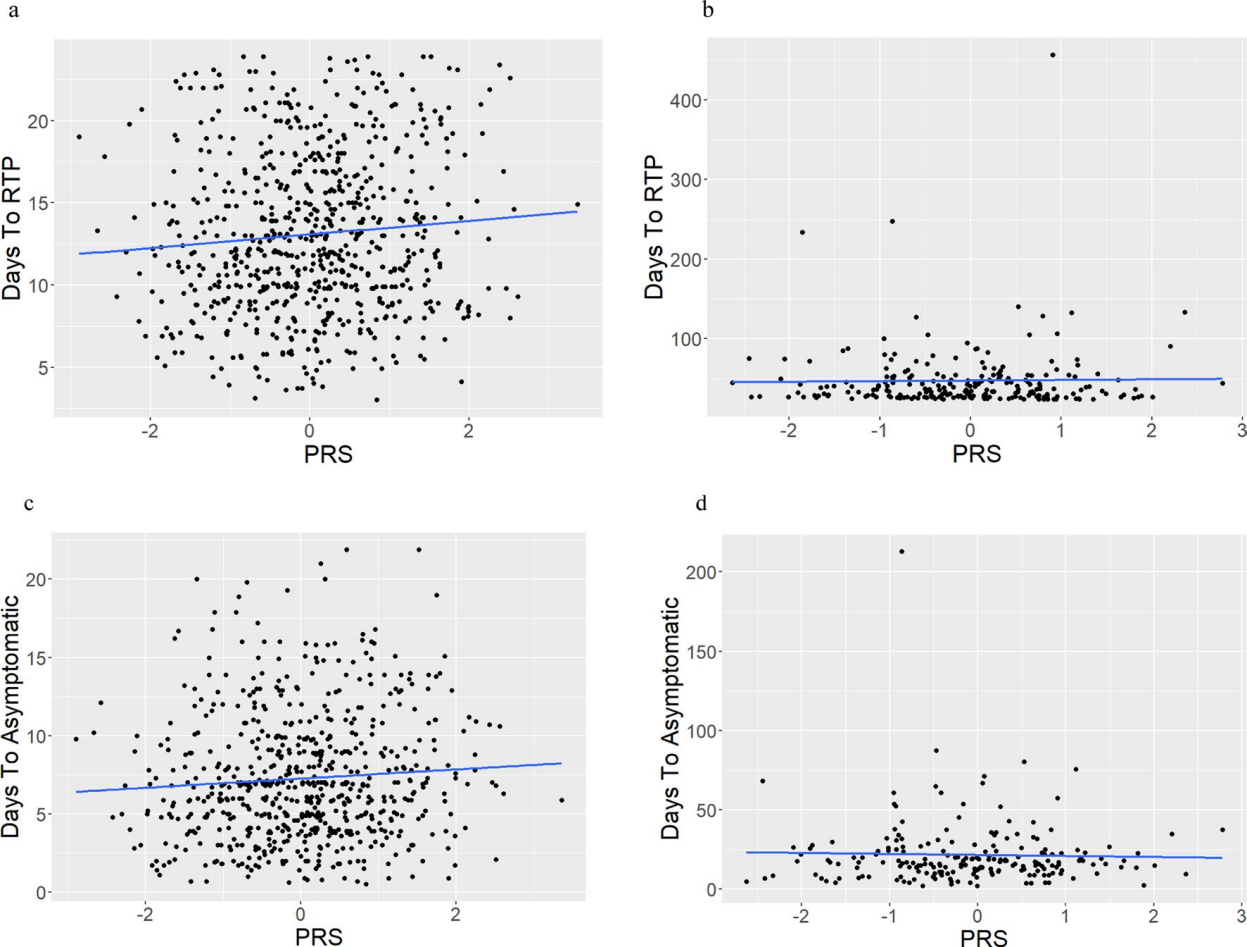
Concussion recovery measures as a function of polygenic risk score (PRS). Number of days to return to play (RTP) by PRS in normal (< 24 days) (**a**) and long (> 24 days) (**b**) RTP categories, and number of days to asymptomatic in normal RTP participants (**c**) and long RTP participants (**d**)

We assessed the number of days a participant took to return to play (injury to RTP interval/RTP) by AD PRS using linear regression (Table 2). We divided the participants into two categories: normal (< 24 days) and long (> 24 days) RTP [54]. We found a relationship between PRS and RTP in the normal RTP category, where higher PRS was associated with a longer injury to RTP interval (*β* = 0.412, standard error [SE] = 0.182, 95% CI 0.055–0.769, *t* value 2.267, *p* = 0.024) (Fig. 2a). There was no relationship in the long RTP category (*p* = 0.778) (Fig. 2b). When we replicated this analysis while covarying for genetic ancestry, sex, LOC status, and military versus civilian origin, the relationship between PRS and RTP in the normal RTP category was no longer significant (*p* = 0.054) (Supplementary Table 3).

**Table 2:** Summary of outcomes of linear regression analyses

Analysis	Estimate (*β*)	Standard error (SE)	*T* value	*p* Value	*R*^2^
PRS and days to normal RTP (Fig. 2a)	0.412	0.183	2.267	**0.024**	Mult. 0.007 Adj. 0.006
PRS and days to long RTP (Fig. 2b)	0.757	2.682	0.282	0.778	Mult. 0.000 Adj. − 0.004
PRS and days to asymptomatic in normal RTP (Fig. 2c)	0.293	0.157	1.869	0.062	Mult. 0.005 Adj. 0.004
PRS and days to asymptomatic in long RTP (Fig. 2d)	− 0.635	1.458	− 0.435	0.664	Mult. 0.001 Adj. − 0.004
Full data; PRS and BESS (Fig. 3a)	0.169	0.252	0.672	0.502	Mult. 0.001 Adj. − 0.001
Full data; PRS and SAC (Fig. 3b)	0.048	0.080	0.603	0.546	Mult. 0.000 Adj. − 0.001
Full data; PRS and SCATSEV (Fig. 3c)	− 0.356	0.885	− 0.402	0.688	Mult. 0.000 Adj. − 0.002
Full data; PRS and SCATSYMP (Fig. 3d)	− 0.150	0.269	− 0.553	0.580	Mult. 0.001 Adj. − 0.002
EUR; PRS and BESS (Fig. 4a)	0.310	0.293	1.059	0.290	Mult. 0.002 Adj. 0.000
EUR; PRS and normal RTP (Fig. 4b)	0.329	0.204	1.612	0.108	Mult. 0.005 Adj. 0.003
EUR; PRS and long RTP (Fig. 4c)	− 0.548	3.097	− 0.177	0.860	Mult. 0.000 Adj. − 0.006
EUR; PRS and SAC (Fig. 4d)	0.038	0.091	0.423	0.673	Mult. 0.000 Adj. − 0.001
EUR; PRS and SCATSEV (Fig. 4e)	0.356	0.987	0.361	0.718	Mult. 0.000 Adj. − 0.002
EUR; PRS and SCATSYMP (Fig. 4f)	0.093	0.303	0.307	0.759	Mult. 0.000 Adj. − 0.002
AFR; PRS and SCATSYMP (Fig. 5)	− 1.796	0.794	− 2.26	**0.028**	Mult. 0.081 Adj. 0.065
PRS and days to normal RTP, F LOC+ (Supplementary Fig. 2a)	− 1.795	3.111	− 0.577	0.576	Mult. 0.029 Adj. − 0.059
PRS and days to normal RTP, F LOC− (Supplementary Fig. 2b)	0.554	0.301	1.841	0.067	Mult. 0.014 Adj. 0.010
PRS and days to normal RTP, M LOC+ (Supplementary Fig. 2c)	0.793	1.090	0.727	0.477	Mult. 0.030 Adj. − 0.027
PRS and days to normal RTP, M LOC− (Supplementary Fig. 2d)	0.329	0.233	1.409	0.16	Mult. 0.005 Adj. 0.002
Normal RTP; PRS and BESS (Supplementary Fig. 3a)	0.153	0.277	0.553	0.58	Mult. 0.001 Adj. − 0.001
Normal RTP; PRS and SAC (Supplementary Fig. 3b)	− 0.007	0.094	− 0.079	0.937	Mult. 0.000 Adj. − 0.001
Normal RTP; PRS and SCATSEV (Supplementary Fig. 3c)	0.308	0.955	0.324	0.746	Mult. 0.003 Adj. − 0.003
Normal RTP; PRS and SCATSYMP (Supplementary Fig. 3d)	0.012	0.297	0.039	0.969	Mult. 0.000 Adj. − 0.003
Long RTP; PRS and BESS (Supplementary Fig. 4a)	0.266	0.576	0.461	0.645	Mult. 0.001 Adj. − 0.004
Long RTP; PRS and SAC (Supplementary Fig. 4b)	0.235	0.149	1.573	0.117	Mult. 0.011 Adj. 0.007
Long RTP; PRS and SCATSEV (Supplementary Fig. 4c)	− 1.397	1.906	− 0.733	0.465	Mult. 0.005 Adj. − 0.004
Long RTP; PRS and SCATSYMP (Supplementary Fig. 4d)	− 0.291	0.523	− 0.557	0.578	Mult. 0.003 Adj. − 0.006
AFR; PRS and BESS (Supplementary Fig. 5a)	− 0.849	0.605	− 1.402	0.163	Mult. 0.015 Adj. 0.008
AFR; PRS and normal RTP (Supplementary Fig. 5b)	0.645	0.523	1.232	0.221	Mult. 0.015 Adj. 0.005
AFR; PRS and long RTP (Supplementary Fig. 5c)	3.203	4.136	0.774	0.446	Mult. 0.023 Adj. − 0.016
AFR; PRS and SAC (Supplementary Fig. 5d)	0.085	0.232	0.366	0.715	Mult. 0.001 Adj. − 0.007
AFR; PRS and SCATSEV (Supplementary Fig. 5e)	− 4.026	2.719	− 1.481	0.144	Mult. 0.036 Adj. 0.020

Bolded values indicate that the results met statistical significance at *p* < 0.05; Mult. = multiple, Adj. = adjusted

*PRS* polygenic risk score, *RTP* return to play, *BESS* balance error scoring system, *SAC* standardized assessment of concussion, *SCATSEV* symptom severity score on the sport concussion assessment tool (SCAT), *SCATSYMP* total number of symptoms score on the SCAT, *EUR* participants of European genetic ancestry, *AFR* participants of African genetic ancestry, *F LOC+* female participants with loss of consciousness (LOC), *F LOC−* female participants without LOC, *M LOC+* male participants with LOC, *M LOC−* male participants without LOC

Since there was a significant relationship between PRS and RTP in the whole normal RTP group, we analyzed the injury to RTP interval by PRS in each participant subgroup (Supplementary Fig. 2a–d). There was no relationship in the F LOC+ group (*p* = 0.576), the M LOC+ group (*p* = 0.477), or the M LOC− group (*p* = 0.16), and the F LOC- group approached significance (*p* = 0.067) (Table 2).

We then assessed the injury to asymptomatic interval as a function of PRS with participants split into normal and long RTP subsets (Table 2). This was done rather than splitting based on the normal injury to asymptomatic interval (< 14 days, as defined previously [43]) so that the results could be directly compared with the RTP analysis. The relationship approached significance in the normal RTP group (*p* = 0.062) (Fig. 2c), but was not significant in the long RTP group (*p* = 0.664) (Fig. 2d).

### Concussion Severity Outcomes as a Function of PRS

**Fig. 3:**
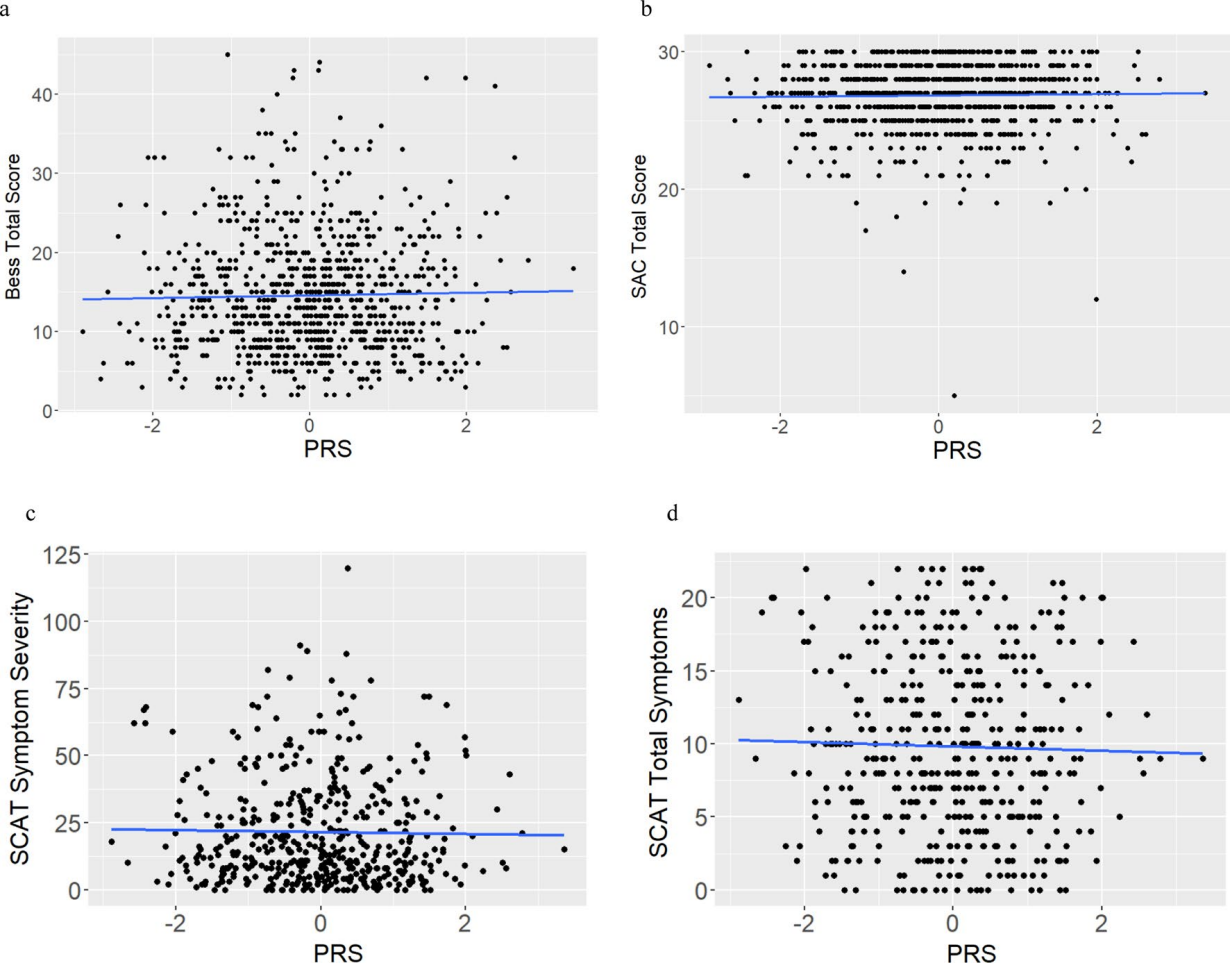
Alzheimer’s disease (AD) polygenic risk score (PRS) and severity outcome measures in the full dataset (Balance error scoring system (BESS) (**a**) and standardized assessment of concussion (SAC) (**b**) total scores, and sport concussion assessment tool (SCAT) symptom severity score (SCATSEV) (**c**) and total number of symptoms (SCATSYMP) (**d**))

Linear regressions were used to assess the impact of AD PRS on concussion severity outcomes (BESS and SAC total scores, SCAT 5 symptom severity score (SCATSEV) and total number of symptoms (SCATSYMP)) (Table 2). No significant relationships were identified in the full data (BESS *p* = 0.502, SAC *p* = 0.546, SCATSEV *p* = 0.688, SCATSYMP *p* = 0.580) (Fig. 3a–d)*.*

Similarly, there were no associations in the normal RTP subset (BESS *p* = 0.580, SAC *p* = 0.937, SCATSEV *p* = 0.746, SCATSYMP *p* = 0.969) (Supplementary Fig. 3a–d) (Table 2). There were also no significant relationships in the long RTP subset (BESS *p* = 0.645, SAC *p* = 0.117, SCATSEV *p* = 0.465, SCATSYMP *p* = 0.578) (Supplementary Fig. 4a–d) (Table 2).

### Concussion Recovery and Severity Across *APOE* Genotypes

We used ANOVA tests to assess concussion recovery and severity by *APOE* genotype (ε3/ε3 versus ε2 carriers, ε3/ε3 versus ε4 carriers) (Table 3). Participants were divided based on whether they were a military or civilian participant. There were no differences between ε3/ε3 participants compared with ε2 carriers or ε4 carriers in either the military or civilian subgroups. There were also no significant differences when this analysis was repeated in only participants of European genetic ancestry (Table 3). Upon replicating these tests with covariates of genetic ancestry, sex, LOC status, and military versus civilian origin, there were no significant differences (Supplementary Table 4).

**Table 3:** Results from ANOVAs of outcomes by apolipoprotein E (*APOE)* genotype (ε3/ε3 versus ε2 or ε4 carriers), participant type (military or civilian origin), and in participants of European (EUR) genetic ancestry

Analysis	*n* ε3/ε3	*n* ε2 or ε4	*p* value
*APOE ε3/ε3* versus ε2 carriers; military versus civilian			
BESS; civilian	397	74	0.646
BESS; military	131	27	0.963
Days to RTP; civilian; normal RTP (< 24 days)	316	58	0.174
Days to RTP; civilian; long RTP (> 24 days)	98	22	0.383
Days to RTP; military; normal RTP	101	19	0.839
Days to RTP; military; long RTP	33	10	0.226
SAC; civilian	407	80	0.441
SAC; military	134	29	0.372
SCATSEV; civilian	249	50	0.140
SCATSEV; military	31	5	0.744
SCATSYMP; civilian	249	50	0.445
SCATSYMP; military	31	5	0.774
*APOE* ε3/ε3 versus ε4 carriers; military versus civilian			
BESS; civilian	397	154	0.074
BESS; military	131	76	0.097
Days to RTP; civilian; normal RTP (< 24 days)	316	129	0.932
Days to RTP; civilian; long RTP (> 24 days)	98	36	0.876
Days to RTP; military; normal RTP (< 24 days)	101	54	0.891
Days to RTP; military; long RTP (> 24 days)	33	24	0.545
SAC; civilian	407	161	0.646
SAC; military	134	78	0.353
SCATSEV; civilian	249	102	0.743
SCATSEV; military	31	21	0.216
SCATSYMP; civilian	249	102	0.943
SCATSYMP; military	31	21	0.181
ε3/ε3 versus ε2 or ε4; EUR ancestry only			
BESS ε3/ε3 versus ε2	423	82	0.767
BESS ε3/ε3 versus ε4	423	169	0.670
Days to RTP ε3/ε3 versus ε2; normal RTP (< 24 days)	336	61	0.548
Days to RTP ε3/ε3 versus ε2; long RTP (> 24 days)	104	27	0.986
Days to RTP ε3/ε3 versus ε4; normal RTP (< 24 days)	336	133	0.613
Days to RTP ε3/ε3 versus ε4; long RTP (> 24 days)	104	47	0.225
SAC ε3/ε3 versus ε2	435	88	0.801
SAC ε3/ε3 versus ε4	435	175	0.700
SCATSEV ε3/ε3 versus ε2	229	45	0.057
SCATSEV ε3/ε3 versus ε4	229	93	0.222
SCATSYMP ε3/ε3 versus ε2	229	45	0.624
SCATSYMP ε3/ε3 versus ε4	229	93	0.193

*BESS* balance error scoring system, *RTP* return to play, *SAC* standardized assessment of concussion, *SCATSEV* symptom severity score on the sport concussion assessment tool (SCAT), *SCATSYMP* total number of symptoms score on the SCAT, *EUR* participants of European genetic ancestry

### Frequency of Long Versus Normal RTP by *APOE* Genotype

We performed a chi-square analysis of RTP category by *APOE* genotype in military and civilian participants to test whether the frequency with which participants fell into the long RTP category was associated with *APOE* genotype. There were no significant findings in either civilian (ε3/ε3 versus ε2: *χ*^2^ (1, *N* = 494) = 0.534, *p* = 0.465; ε3/ε3 versus ε4: *χ*^2^ (1, *N* = 579) = 0.228, *p* = 0.633) or military (ε3/ε3 versus ε2: *χ*^2^ (1, *N* = 163) = 1.192, *p* = 0.275; ε3/ε3 versus ε4: *χ*^2^ (1, *N* = 212) = 0.946, *p* = 0.331) participants.

### Outcomes by PRS in European and African Genetic Ancestry

**Fig. 4:**
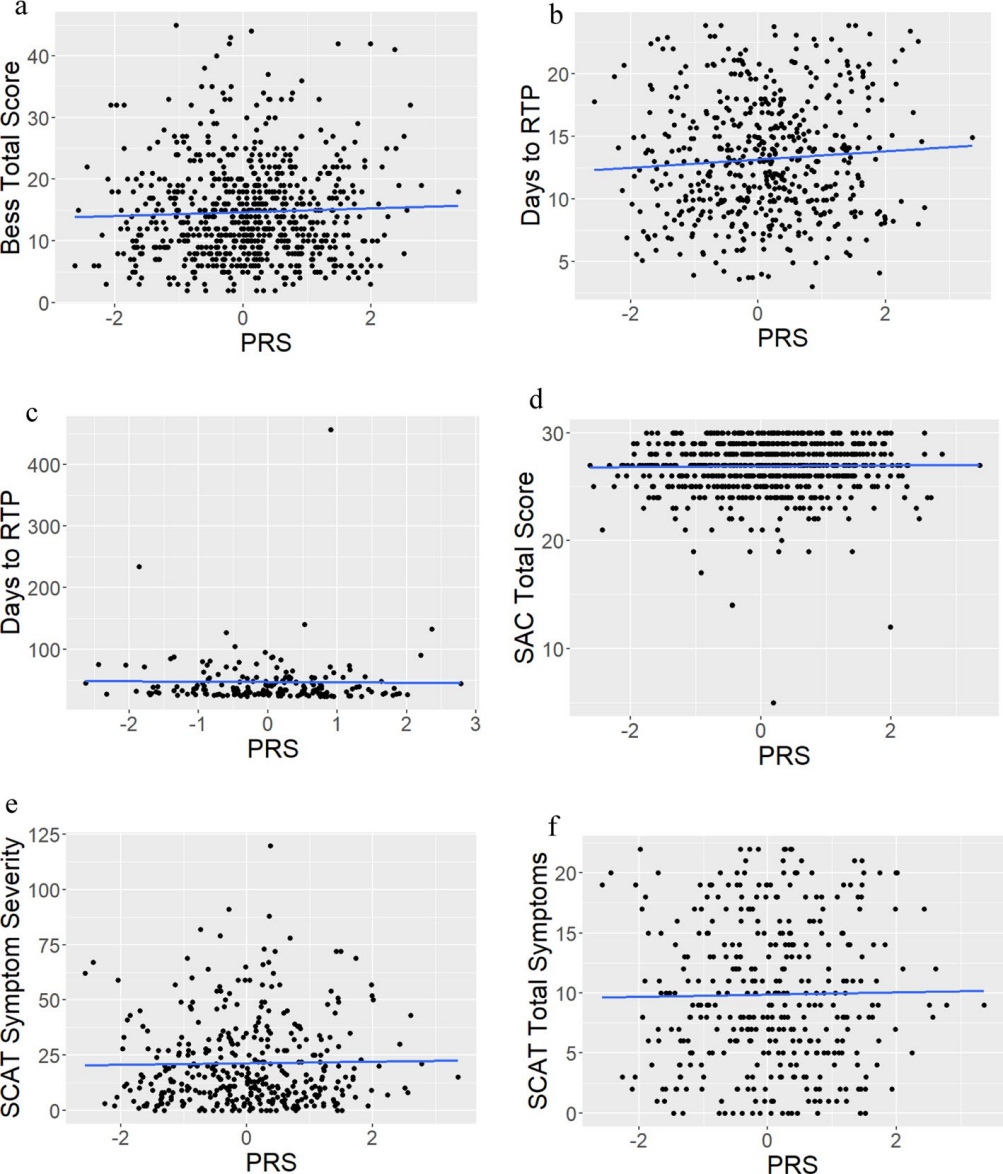
Outcome measures (balance error scoring system (BESS) total score (**a**), days to return to play (RTP) in normal (< 24 days) subset (**b**), days to RTP in long (> 24 days) subset (**c**), total score on standardized assessment of concussion (SAC) (**d**), sport concussion assessment tool (SCAT) symptom severity score (**e**), and SCAT total number of symptoms (**f**)) as a function of Alzheimer’s disease (AD) polygenic risk score (PRS) in participants with European genetic ancestry

**Fig. 5:**
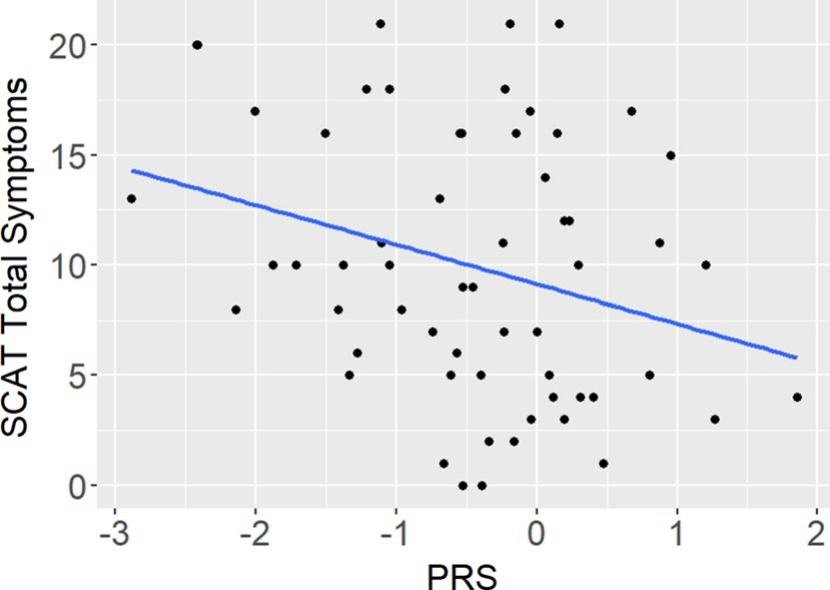
In individuals with African genetic ancestry, higher Alzheimer’s disease (AD) polygenic risk score (PRS) was associated with lower sport concussion assessment tool (SCAT) total number of symptoms (SCATSYMP) (*p* = 0.0276)

The selected PRS was derived entirely from individuals of European ancestry, but the CARE dataset is ethnically and racially heterogeneous [21]. As such, we performed a subgroup analysis in the CARE participants of European ancestry, as the European-derived PRS may translate better to this subgroup than participants of other ancestries. We, therefore, used linear regressions to look for associations of PRS and outcome measures with participants divided based on genetic ancestry (African or European). There were no relationships in European participants only (BESS *p* = 0.290; normal RTP *p* = 0.108; long RTP *p* = 0.860; SAC *p* = 0.673; SCATSEV *p* = 0.718; SCATSYMP *p* = 0.759) (Fig. 4a–f). In individuals with African genetic ancestry, higher AD PRS was associated with lower SCAT total number of symptoms (SCATSYMP) (*β* =  − 1.796, SE 0.794, 95% CI − 3.386 to − 0.205], *t* ratio − 2.260, *p* = 0.028) (Fig. 5). There were no other significant differences (BESS *p* = 0.163, normal RTP *p* = 0.221, long RTP *p* = 0.446, SAC *p* = 0.715, SCATSEV *p* = 0.144) (Supplementary Fig. 5a–e).

## Discussion

Our preliminary results identified that an increase in AD PRS was associated with a slight increase to the injury to RTP interval in participants who took 24 days or less to RTP. However, as the remaining analyses and metrics were generally non-significant, and this difference was no longer significant with the addition of covariates, our findings generally indicate that NCAA student athletes and Military Service Academy students with high genetic risk for AD are unlikely to experience significantly worse concussions or poorer recovery than those with lower genetic risk.

Participants in the F LOC− group were 0.35 years (approximately 4.2 months) younger than participants in the M LOC− group. However, though this was statistically significant, a 4-month age difference is unlikely to be clinically meaningful. Similarly, there was little evidence for an initial difference in injury or symptom severity between the groups. While the F LOC− group took longer to become asymptomatic than the M LOC− group, and the M LOC+ group demonstrated fewer symptoms on the SCAT5 than the F LOC− group, there was no consistent pattern of findings that suggested worse initial injury severity or recovery trajectories between the groups. Similarly, significant chi-square tests of both self-reported race/ethnicity and genetically derived ancestry are likely related to demographic differences in sports participation in the CARE Consortium, as described previously [73]. These significant tests are unlikely to represent clinically meaningful differences between groups on concussion severity and/or recovery.

Intriguingly, though the 21 *APOE* ε2/ε4 participants were excluded, 20 of these participants belonged to the M LOC− group while only one belonged to the F LOC− group. This is curious given that the *APOE* gene is located on chromosome 19 and is, thus, not expected to exhibit sex differences in prevalence. Furthermore, it was surprising that we found no associations between *APOE* genotype and concussion severity/recovery. Multiple previous reports have found *APOE* ε4 to be associated with poorer concussion outcomes [26–33]; however, this is not a definitive relationship, as other studies have not identified a clear influence of *APOE* ε4 on either injury severity or recovery [74, 75]. Our null finding may be a consequence of low power but could also indicate our chosen recovery/severity metrics are not sensitive to identify *APOE*-associated differences in concussion severity and/or recovery. However, replications are needed to further explore these possibilities.

We observed a relationship between PRS and the injury to RTP interval in participants who took 24 days or less to return to play after an injury where higher AD PRS was associated with elevated recovery time. For every 1 standard deviation increase in PRS, the injury to RTP interval increased by 0.4121 days (9.89 h). However, this relationship was no longer significant when the model was replicated with the addition of covariates for genetic ancestry, LOC status, sex, and military versus civilian participant origin. Furthermore, the adjusted *R*^2^ value of the regression between AD PRS and the injury to RTP interval was 0.00585, indicating AD PRS only explained 0.585% of the variability in RTP. Additionally, there was no relationship between AD PRS and either the injury to asymptomatic or RTP intervals in the long (> 24 days) RTP category. However, the long RTP group had fewer datapoints and numerous extremely long (100+ days) injury to RTP and/or asymptomatic intervals, both of which may partly contribute to the null finding in that group. Last, upon subgroup analyses based on sex and LOC status of the injury to RTP interval within the normal RTP group, there were no significant relationships. Together, our results do not provide compelling evidence for an association between AD PRS and concussion recovery metrics.

Upon subgroup analyses based on genetic ancestry, we observed that higher PRS was associated with a lower SCAT total number of symptoms in individuals with African genetic ancestry, which was intriguing given that we would have expected higher AD genetic risk to be associated with more concussion symptoms. However, the *R*^2^ value was very small (multiple *R*^2^ = 0.081, adjusted *R*^2^ = 0.0651), indicating very little variance in the number of concussion symptoms can be attributed to PRS. Additionally, since the PRS was derived from European participants only, this finding in African genetic ancestry participants must be interpreted with significant caution.

Along these lines, the main limitation of our study is that the selected PRS may not have been an optimal choice. We primarily chose this score because it was generated from a large sample size, but this population was entirely European [52]. The CARE dataset is not homogeneous (Table 1) [21], and prior reports have identified poor transferability of European scores into African ancestry populations [76, 77]. However, as we could not identify a PRS score that was reflective of the CARE demographics, we performed subgroup analyses based on ancestry to assess the PRS in CARE participants of European ancestry only, as well as the transferability of the chosen PRS to diverse CARE participants. The lack of significant associations in the European ancestry subsample suggests that the selected PRS was acceptable for use in this study, though these analyses should ideally be replicated using a PRS more reflective of the CARE sample once such a score is available.

Another limitation is that the injury to RTP interval does not capture all dimensions of concussion recovery. Our method assumes that the interval is solely reflective of continued concussion symptomology, which may be an incorrect assumption in some cases. Recovery is a multifactorial process, and many variables that can affect recovery trajectories were not considered here. For example, if an athlete suffered a simultaneous concussion and musculoskeletal injury, the injury to RTP interval could be influenced by the musculoskeletal injury, but we would be unable to distinguish this. Also, we did not consider psychological effects on recovery, but this is a critical future direction. Previous reports have identified relationships between concussion and psychological health in the form of anxiety/depression [78–83], particularly in individuals with persistent postconcussive symptoms [84–87]. Additionally, somatization is the biggest factor influencing concussion recovery [88]. As such, future studies would benefit by incorporating psychological test scores alongside physical recovery measures when assessing concussion recovery/severity in the context of genetic risk for neurodegenerative disease. An important future direction will be investigating PRS-associated neuroimaging changes in this cohort, such as white matter (WM) damage using diffusion tensor imaging (DTI). Reports from the CARE Consortium and other studies have noted WM changes, such as worsened myelin integrity, in concussed athletes/military service members [89–95], but few studies have examined whether concussion-associated WM damage is exacerbated in individuals with high genetic risk for neurodegenerative disease. One study found limited evidence for altered fractional anisotropy (FA) in the cingulum of *APOE* ɛ4 carriers with concussion/mild traumatic brain injury [96], while another observed that military veterans who are *APOE* ɛ4 carriers may be more vulnerable to WM abnormalities after blast exposure [97]. This evidence is supportive of a link between AD genetic risk and WM changes after concussion, but fewer explorations into lesser-known AD risk genes using summary tools such as PRS have been reported.

Finally, though we excluded repeated injuries from this analysis, investigating the effects of AD genetic risk on recovery after multiple injuries is an important next step. Though we did not identify consistent links between AD genetic risk and recovery/severity in the first injury exposure, it is possible that AD genetic risk may be more strongly associated with severity/recovery in the context of multiple injuries. We will investigate this possibility in future studies. Exploration into potential effects of subconcussive injuries may also offer intriguing information particularly in the context of intersport differences in head injury exposure. However, it may prove challenging to establish protocols and standards to facilitate the capture of data on subconcussive and/or asymptomatic brain injuries.

## Conclusions

W observed an association of AD PRS with lengthened concussion recovery time based on one metric in collegiate athletes and military service academy students who were concussed during the CARE study. However, the signal was attenuated with inclusion of covariates, and no additional analyses or metrics reached statistical significance. It is, therefore, unlikely that AD genetic risk is strongly associated with concussion severity or recovery in this sample. Future studies should attempt to replicate these analyses in larger samples with longer follow-up using PRS calculated from multiple/diverse populations to clarify and add to these findings. Additionally, incorporating multiple modulators and metrics of injury recovery, including psychological health, coincident non-concussion injuries, and neuroimaging metrics such as diffusion tensor imaging, will be of significant value. Finally, investigation of subconcussive injuries will also be necessary to better understand injury incidence rates across different sports.

## Supplementary Information

Below is the link to the electronic supplementary material.

Supplementary file1 (DOCX 500 KB)

## Declarations

### Data availability statement

Patient data are accessible as part of CARE 1.0 on the Federal Interagency Traumatic Brain Injury Research Informatics System (FITBIR, ftbir.nih.gov). Criteria for access are determined by each of the entities hosting the data and the governance of their respective regulatory committees.

### Code availability

Code is available upon request.

## References

[1] Langlois JA, Rutland-Brown W, Wald MM. The epidemiology and impact of traumatic brain injury: a brief overview. J Head Trauma Rehabil. 2006;21(5):375–8. 16983222 10.1097/00001199-200609000-00001

[2] Giza CC, Kutcher JS. An introduction to sports concussions. Continuum (Minneap Minn). 2014;20(6 Sports Neurology):1545–51. 25470159 10.1212/01.CON.0000458975.78766.11 PMC4274166

[3] Daneshvar DH, et al. The epidemiology of sport-related concussion. Clin Sports Med. 2011;30(1):1–17, vii. 21074078 10.1016/j.csm.2010.08.006 PMC2987636

[4] Wasserman EB, et al. Academic dysfunction after a concussion among US high school and college students. Am J Public Health. 2016;106(7):1247–53. 27196651 10.2105/AJPH.2016.303154 PMC4984778

[5] Patricios JS, et al. Consensus statement on concussion in sport: the 6th International conference on Concussion in Sport-Amsterdam, October 2022. Br J Sports Med. 2023;57(11):695–711. 37316210 10.1136/bjsports-2023-106898

[6] Halstead ME, Walter KD, American Academy of Pediatrics. Clinical report—sport-related concussion in children and adolescents. Pediatrics. 2010;126(3):597–615. 20805152 10.1542/peds.2010-2005

[7] Halstead ME, et al. Returning to learning following a concussion. Pediatrics. 2013;132(5):948–57. 24163302 10.1542/peds.2013-2867

[8] McCrory P, Davis G, Makdissi M. Second impact syndrome or cerebral swelling after sporting head injury. Curr Sports Med Rep. 2012;11(1):21–3. 22236821 10.1249/JSR.0b013e3182423bfd

[9] Bey T, Ostick B. Second impact syndrome. West J Emerg Med. 2009;10(1):6–10. 19561758 PMC2672291

[10] Saunders RL, Harbaugh RE. The second impact in catastrophic contact-sports head trauma. JAMA. 1984;252(4):538–9. 6737652

[11] Cantu RC. Second-impact syndrome. Clin Sports Med. 1998;17(1):37–44. 9475969 10.1016/s0278-5919(05)70059-4

[12] Konrad C, et al. Long-term cognitive and emotional consequences of mild traumatic brain injury. Psychol Med. 2011;41(6):1197–211. 20860865 10.1017/S0033291710001728

[13] McAllister T, McCrea M. Long-term cognitive and neuropsychiatric consequences of repetitive concussion and head-impact exposure. J Athl Train. 2017;52(3):309–17. 28387556 10.4085/1062-6050-52.1.14 PMC5384827

[14] Li Y, et al. Head injury as a risk factor for dementia and Alzheimer’s disease: a systematic review and meta-analysis of 32 observational studies. PLoS ONE. 2017;12(1): e0169650. 28068405 10.1371/journal.pone.0169650 PMC5221805

[15] Livingston G, et al. Dementia prevention, intervention, and care: 2024 report of the Lancet standing Commission. Lancet. 2024;404(10452):572–628. 39096926 10.1016/S0140-6736(24)01296-0

[16] Plassman BL, et al. Documented head injury in early adulthood and risk of Alzheimer’s disease and other dementias. Neurology. 2000;55(8):1158–66. 11071494 10.1212/wnl.55.8.1158

[17] Farrington DP. Longitudinal research strategies: advantages, problems, and prospects. J Am Acad Child Adolesc Psychiatry. 1991;30(3):369–74. 2055872 10.1097/00004583-199105000-00003

[18] Caruana EJ, et al. Longitudinal studies. J Thorac Dis. 2015;7(11):E537–40. 26716051 10.3978/j.issn.2072-1439.2015.10.63 PMC4669300

[19] Raman R, et al. Tackling a major deficiency of diversity in Alzheimer’s disease therapeutic trials: an CTAD task force report. J Prev Alzheimers Dis. 2022;9(3):388–92. 35841239 10.14283/jpad.2022.50 PMC9098373

[20] Mindt MR, et al. Improving generalizability and study design of Alzheimer’s disease cohort studies in the United States by including under-represented populations. Alzheimers Dement. 2023;19(4):1549–57. 36372959 10.1002/alz.12823 PMC10101866

[21] Broglio SP, et al. A national study on the effects of concussion in collegiate athletes and US Military Service Academy members: the NCAA-DoD Concussion Assessment, Research and Education (CARE) Consortium structure and methods. Sports Med. 2017;47(7):1437–51. 28281095 10.1007/s40279-017-0707-1 PMC5488134

[22] Teasdale GM, et al. Association of apolipoprotein E polymorphism with outcome after head injury. Lancet. 1997;350(9084):1069–71. 10213549 10.1016/S0140-6736(97)04318-3

[23] Hellstrøm T, et al. APOE-ε4 is associated with reduced verbal memory performance and higher emotional, cognitive, and everyday executive function symptoms two months after mild traumatic brain injury. Front Neurol. 2022;13: 735206. 35250800 10.3389/fneur.2022.735206 PMC8888909

[24] Sundström A, et al. APOE influences on neuropsychological function after mild head injury: within-person comparisons. Neurology. 2004;62(11):1963–6. 15184597 10.1212/01.wnl.0000129268.83927.a8

[25] Sundström A, et al. Fatigue before and after mild traumatic brain injury: pre-post-injury comparisons in relation to Apolipoprotein E. Brain Inj. 2007;21(10):1049–54. 17891567 10.1080/02699050701630367

[26] Yue JK, et al. Apolipoprotein E epsilon 4 (APOE-ε4) genotype is associated with decreased 6-month verbal memory performance after mild traumatic brain injury. Brain Behav. 2017;7(9): e00791. 28948085 10.1002/brb3.791 PMC5607554

[27] Merritt VC, et al. Apolipoprotein E (APOE) ε4 genotype is associated with reduced neuropsychological performance in military veterans with a history of mild traumatic brain injury. J Clin Exp Neuropsychol. 2018;40(10):1050–61. 30124361 10.1080/13803395.2018.1508555

[28] Liberman JN, et al. Apolipoprotein E epsilon 4 and short-term recovery from predominantly mild brain injury. Neurology. 2002;58(7):1038–44. 11940689 10.1212/wnl.58.7.1038

[29] Isoniemi H, et al. Outcome of traumatic brain injury after three decades—relationship to ApoE genotype. J Neurotrauma. 2006;23(11):1600–8. 17115907 10.1089/neu.2006.23.1600

[30] Teng Z, et al. ApoE influences the blood-brain barrier through the NF-κB/MMP-9 pathway after traumatic brain injury. Sci Rep. 2017;7(1):6649. 28751738 10.1038/s41598-017-06932-3 PMC5532277

[31] Mahley RW, Huang Y. Apolipoprotein E sets the stage: response to injury triggers neuropathology. Neuron. 2012;76(5):871–85. 23217737 10.1016/j.neuron.2012.11.020 PMC4891195

[32] Lynch JR, et al. APOE Genotype and an ApoE-mimetic peptide modify the systemic and central nervous system inflammatory response. J Biol Chem. 2003;278(49):48529–33. 14507923 10.1074/jbc.M306923200

[33] Horsburgh K, et al. The role of apolipoprotein E in Alzheimer’s disease, acute brain injury and cerebrovascular disease: evidence of common mechanisms and utility of animal models. Neurobiol Aging. 2000;21(2):245–55. 10867209 10.1016/s0197-4580(00)00097-x

[34] Harris FM, et al. Carboxyl-terminal-truncated apolipoprotein E4 causes Alzheimer’s disease-like neurodegeneration and behavioral deficits in transgenic mice. Proc Natl Acad Sci. 2003;100(19):10966–71. 12939405 10.1073/pnas.1434398100 PMC196910

[35] Guo L, LaDu MJ, Van Eldik LJ. A dual role for apolipoprotein E in neuroinflammation. J Mol Neurosci. 2004;23(3):205–12. 15181248 10.1385/JMN:23:3:205

[36] Giarratana AO, et al. APOE4 genetic polymorphism results in impaired recovery in a repeated mild traumatic brain injury model and treatment with Bryostatin-1 improves outcomes. Sci Rep. 2020;10(1):19919. 33199792 10.1038/s41598-020-76849-x PMC7670450

[37] Liu CC, et al. Apolipoprotein E and Alzheimer disease: risk, mechanisms and therapy. Nat Rev Neurol. 2013;9(2):106–18. 23296339 10.1038/nrneurol.2012.263 PMC3726719

[38] Thal DR, et al. Phases of A beta-deposition in the human brain and its relevance for the development of AD. Neurology. 2002;58(12):1791–800. 12084879 10.1212/wnl.58.12.1791

[39] Braak H, et al. Staging of Alzheimer disease-associated neurofibrillary pathology using paraffin sections and immunocytochemistry. Acta Neuropathol. 2006;112(4):389–404. 16906426 10.1007/s00401-006-0127-z PMC3906709

[40] Bellenguez C, et al. New insights into the genetic etiology of Alzheimer’s disease and related dementias. Nat Genet. 2022;54(4):412–36. 35379992 10.1038/s41588-022-01024-z PMC9005347

[41] Lewis CM, Vassos E. Polygenic risk scores: from research tools to clinical instruments. Genome Med. 2020;12(1):44. 32423490 10.1186/s13073-020-00742-5 PMC7236300

[42] Choi SW, Mak TS-H, O’Reilly PF. Tutorial: a guide to performing polygenic risk score analyses. Nat Protoc. 2020;15(9):2759–72. 32709988 10.1038/s41596-020-0353-1 PMC7612115

[43] Ibanez L, et al. Polygenic risk scores in neurodegenerative diseases: a review. Curr Genet Med Rep. 2019;7(1):22–9.

[44] Purcell S, et al. PLINK: a tool set for whole-genome association and population-based linkage analyses. Am J Hum Genet. 2007;81(3):559–75. 17701901 10.1086/519795 PMC1950838

[45] Bretzin AC, et al. Association of sex with adolescent soccer concussion incidence and characteristics. JAMA Netw Open. 2021;4(4): e218191. 33904911 10.1001/jamanetworkopen.2021.8191 PMC8080231

[46] Covassin T, Moran R, Elbin RJ. Sex differences in reported concussion injury rates and time loss from participation: an update of the National Collegiate Athletic Association Injury Surveillance Program from 2004–2005 through 2008–2009. J Athl Train. 2016;51(3):189–94. 26950073 10.4085/1062-6050-51.3.05 PMC4852524

[47] Bretzin AC, et al. Sex differences in the clinical incidence of concussions, missed school days, and time loss in high school student-athletes: part 1. Am J Sports Med. 2018;46(9):2263–9. 29879362 10.1177/0363546518778251

[48] Iverson GL, et al. Examining whether loss of consciousness is associated with worse performance on the SCAT5 and slower clinical recovery after concussion in professional athletes. J Neurotrauma. 2022;40(21–22):2330–40. 10.1089/neu.2022.0043 36541353

[49] McCrea M, et al. Incidence, clinical course, and predictors of prolonged recovery time following sport-related concussion in high school and college athletes. J Int Neuropsychol Soc. 2013;19(1):22–33. 23058235 10.1017/S1355617712000872

[50] Auton A, et al. A global reference for human genetic variation. Nature. 2015;526(7571):68–74. 26432245 10.1038/nature15393 PMC4750478

[51] Sudmant PH, et al. An integrated map of structural variation in 2,504 human genomes. Nature. 2015;526(7571):75–81. 26432246 10.1038/nature15394 PMC4617611

[52] de Rojas I, et al. Common variants in Alzheimer’s disease and risk stratification by polygenic risk scores. Nat Commun. 2021;12(1):3417. 34099642 10.1038/s41467-021-22491-8 PMC8184987

[53] D’Lauro C, et al. Reconsidering return-to-play times: a broader perspective on concussion recovery. Orthop J Sports Med. 2018;6(3):2325967118760854. 29568786 10.1177/2325967118760854 PMC5858632

[54] McAllister TW, et al. Characteristics and outcomes of athletes with slow recovery from sports-related concussion: A CARE consortium study. Neurology. 2023;100(14):e1510–9. 36653178 10.1212/WNL.0000000000206853 PMC10104617

[55] McCrory P, et al. Consensus statement on Concussion in Sport 3rd International conference on Concussion in Sport held in Zurich, November 2008. Clin J Sport Med. 2009;19(3):185–200. 19423971 10.1097/JSM.0b013e3181a501db

[56] McCrory P, et al. Consensus statement on concussion in sport-the 5(th) international conference on concussion in sport held in Berlin, October 2016. Br J Sports Med. 2017;51(11):838–47. 28446457 10.1136/bjsports-2017-097699

[57] McCrory P, et al. Consensus statement on concussion in sport: the 4th International conference on Concussion in Sport, Zurich, November 2012. J Athl Train. 2013;48(4):554–75. 23855364 10.4085/1062-6050-48.4.05 PMC3715021

[58] McCrory P, et al. Summary and agreement statement of the 2nd International conference on Concussion in Sport, Prague 2004. Br J Sports Med. 2005;39(4):196–204. 15793085 10.1136/bjsm.2005.018614 PMC1725173

[59] Aubry M, et al. Summary and agreement statement of the First International conference on Concussion in Sport, Vienna 2001 Recommendations for the improvement of safety and health of athletes who may suffer concussive injuries. Br J Sports Med. 2002;36(1):6–10. 11867482 10.1136/bjsm.36.1.6 PMC1724447

[60] Broglio SP, et al. The natural history of sport-related concussion in collegiate athletes: findings from the NCAA-DoD CARE Consortium. Sports Med. 2022;52(2):403–15. 34427877 10.1007/s40279-021-01541-7

[61] Collins MW, et al. Cumulative effects of concussion in high school athletes. Neurosurgery. 2002;51(5):1175–9 (**discussion 1180–1181**). 12383362 10.1097/00006123-200211000-00011

[62] Collins MW, et al. Relationship between postconcussion headache and neuropsychological test performance in high school athletes. Am J Sports Med. 2003;31(2):168–73. 12642248 10.1177/03635465030310020301

[63] Koerte IK, et al. Sex-related differences in the effects of sports-related concussion: a review. J Neuroimaging. 2020;30(4):387–409. 32533752 10.1111/jon.12726 PMC8221087

[64] McGroarty NK, Brown SM, Mulcahey MK. Sport-related concussion in female athletes: a systematic review. Orthop J Sports Med. 2020;8(7):2325967120932306. 32728590 10.1177/2325967120932306 PMC7366411

[65] Brett BL, et al. Investigating the range of symptom endorsement at initiation of a graduated return-to-play protocol after concussion and duration of the protocol: a study from the National Collegiate Athletic Association-Department of Defense Concussion, Assessment, Research, and Education (CARE) Consortium. Am J Sports Med. 2020;48(6):1476–84. 32298132 10.1177/0363546520913252

[66] Echemendia RJ, et al. The Sport Concussion Assessment Tool 5th edition (SCAT5): background and rationale. Br J Sports Med. 2017;51(11):848–50. 28446453 10.1136/bjsports-2017-097506

[67] Petit KM, et al. The Sport Concussion Assessment Tool-5 (SCAT5): Baseline Assessments in NCAA Division I Collegiate Student-Athletes. Int J Exerc Sci. 2020;13(3):1143–55. 32922635 10.70252/SUJD2658 PMC7449330

[68] Bell DR, et al. Systematic review of the balance error scoring system. Sports Health. 2011;3(3):287–95. 23016020 10.1177/1941738111403122 PMC3445164

[69] Riemann BL, Guskiewicz KM. Effects of mild head injury on postural stability as measured through clinical balance testing. J Athl Train. 2000;35(1):19–25. 16558603 PMC1323433

[70] McCrea M, et al. Acute effects and recovery time following concussion in collegiate football players: the NCAA Concussion Study. JAMA. 2003;290(19):2556–63. 14625332 10.1001/jama.290.19.2556

[71] Guskiewicz KM, Ross SE, Marshall SW. Postural stability and neuropsychological deficits after concussion in collegiate athletes. J Athl Train. 2001;36(3):263–73. 12937495 PMC155417

[72] McCrea M, et al. Standardized assessment of concussion (SAC): on-site mental status evaluation of the athlete. J Head Trauma Rehabil. 1998;13(2):27–35. 9575254 10.1097/00001199-199804000-00005

[73] Boltz AJ, et al. Intersection of race and socioeconomic status on concussion recovery among NCAA student-athletes: a CARE Consortium Study. Med Sci Sports Exerc. 2023;55(12):2180–93. 37486776 10.1249/MSS.0000000000003258

[74] Padgett CR, Summers MJ, Skilbeck CE. Is APOE ε4 associated with poorer cognitive outcome following traumatic brain injury? A meta-analysis. Neuropsychology. 2016;30(7):775–90. 26986748 10.1037/neu0000270

[75] Shadli RM, et al. APOE genotype and neuropsychological outcome in mild-to-moderate traumatic brain injury: a pilot study. Brain Inj. 2011;25(6):596–603. 21534737 10.3109/02699052.2011.572947

[76] Kamiza AB, et al. Transferability of genetic risk scores in African populations. Nat Med. 2022;28(6):1163–6. 35654908 10.1038/s41591-022-01835-x PMC9205766

[77] Duncan L, et al. Analysis of polygenic risk score usage and performance in diverse human populations. Nat Commun. 2019;10(1):3328. 31346163 10.1038/s41467-019-11112-0 PMC6658471

[78] Broshek DK, De Marco AP, Freeman JR. A review of post-concussion syndrome and psychological factors associated with concussion. Brain Inj. 2015;29(2):228–37. 25383595 10.3109/02699052.2014.974674

[79] Schmidt JD, et al. Medical disqualification following concussion in collegiate student-athletes: findings from the CARE Consortium. Sports Med. 2020;50(10):1843–55. 32557231 10.1007/s40279-020-01302-y

[80] Stein MB, et al. Risk of posttraumatic stress disorder and major depression in civilian patients after mild traumatic brain injury: a TRACK-TBI Study. JAMA Psychiat. 2019;76(3):249–58. 10.1001/jamapsychiatry.2018.4288 PMC6439818 30698636

[81] Iverson GL, Greenberg J, Cook NE. Anxiety is associated with diverse physical and cognitive symptoms in youth presenting to a multidisciplinary concussion clinic. Front Neurol. 2021;12: 811462. 35197916 10.3389/fneur.2021.811462 PMC8858805

[82] Vargas G, et al. Predictors and prevalence of postconcussion depression symptoms in collegiate athletes. J Athl Train. 2015;50(3):250–5. 25643158 10.4085/1062-6050-50.3.02 PMC4477919

[83] Sandel N, et al. Anxiety and mood clinical profile following sport-related concussion: from risk factors to treatment. Sport Exerc Perform Psychol. 2017;6(3):304–23. 29130023 10.1037/spy0000098 PMC5679311

[84] Lambert M, et al. Depressive symptoms in individuals with persistent postconcussion symptoms: a systematic review and meta-analysis. JAMA Netw Open. 2022;5(12): e2248453. 36574246 10.1001/jamanetworkopen.2022.48453 PMC9857135

[85] Ponsford J, et al. Predictors of postconcussive symptoms 3 months after mild traumatic brain injury. Neuropsychology. 2012;26(3):304–13. 22468823 10.1037/a0027888

[86] Mittenberg W, et al. Treatment of post-concussion syndrome following mild head injury. J Clin Exp Neuropsychol. 2001;23(6):829–36. 11910547 10.1076/jcen.23.6.829.1022

[87] Corwin DJ, et al. Characteristics of prolonged concussion recovery in a pediatric subspecialty referral population. J Pediatr. 2014;165(6):1207–15. 25262302 10.1016/j.jpeds.2014.08.034 PMC4253594

[88] Nelson LD, et al. Preinjury somatization symptoms contribute to clinical recovery after sport-related concussion. Neurology. 2016;86(20):1856–63. 27164666 10.1212/WNL.0000000000002679 PMC4873681

[89] Wang JY, et al. Longitudinal changes of structural connectivity in traumatic axonal injury. Neurology. 2011;77(9):818–26. 21813787 10.1212/WNL.0b013e31822c61d7 PMC3162636

[90] Wu YC, et al. Longitudinal white-matter abnormalities in sports-related concussion: a diffusion MRI study. Neurology. 2020;95(7):e781–92. 32641518 10.1212/WNL.0000000000009930 PMC7605507

[91] Mustafi SM, et al. Acute white-matter abnormalities in sports-related concussion: a diffusion tensor imaging study from the NCAA-DoD CARE Consortium. J Neurotrauma. 2018;35(22):2653–64. 29065805 10.1089/neu.2017.5158 PMC6238613

[92] Lancaster MA, et al. Chronic differences in white matter integrity following sport-related concussion as measured by diffusion MRI: 6-Month follow-up. Hum Brain Mapp. 2018;39(11):4276–89. 29964356 10.1002/hbm.24245 PMC6179912

[93] Murugavel M, et al. A longitudinal diffusion tensor imaging study assessing white matter fiber tracts after sports-related concussion. J Neurotrauma. 2014;31(22):1860–71. 24786666 10.1089/neu.2014.3368 PMC4224056

[94] Meier TB, et al. Longitudinal assessment of white matter abnormalities following sports-related concussion. Hum Brain Mapp. 2016;37(2):833–45. 26663463 10.1002/hbm.23072 PMC6867335

[95] Henry LC, et al. Acute and chronic changes in diffusivity measures after sports concussion. J Neurotrauma. 2011;28(10):2049–59. 21864134 10.1089/neu.2011.1836

[96] Hellstrøm T, et al. Apolipoprotein ɛ4 status and brain structure 12 months after mild traumatic injury: brain age prediction using brain morphometry and diffusion tensor imaging. J Clin Med. 2021;10(3):418. 33499167 10.3390/jcm10030418 PMC7865561

[97] Sullivan DR, et al. Close-range blast exposure is associated with altered white matter integrity in apolipoprotein ɛ4 carriers. J Neurotrauma. 2019;36(23):3264–73. 31232163 10.1089/neu.2019.6489

