# Supplementary material for: Association of Alzheimer’s Disease Polygenic Risk Score with Concussion Severity and Recovery Metrics
Source: Sports Med. 2025 Jan 16;55(6):1487–503. doi: 10.1007/s40279-024-02150-w (PMC12152024; doi:10.1007/s40279-024-02150-w)
Supplement: Supplementary file 1 — Supplementary file1 (DOCX 500 KB) [file 40279_2024_2150_MOESM1_ESM.docx]

**Association of Alzheimer’s disease polygenic risk score with concussion severity and recovery metrics**

**Sports Medicine**

Kaitlyn M. Dybing^1, 2, 3*^, Thomas W. McAllister^3, 4, 5^, Yu-Chien Wu^1, 2, 3^, Brenna C. McDonald^1, 2, 3, 4, 5^, Steven P. Broglio^7^, Jason P. Mihalik^8^, Kevin M. Guskiewicz^8^, Joshua T. Goldman^9^, Jonathan C. Jackson^10, 11, 12^, Andrew J. Saykin^1, 2, 3, 4, 5, 6^ , Shannon L. Risacher^1, 2, 3*#^, & Kelly N. H. Nudelman^2, 6#^

*^1^Department of Radiology & Imaging Sciences, Indiana University School of Medicine, Indianapolis, IN, USA.*

*^2^Indiana Alzheimer’s Disease Research Center, Indiana University School of Medicine, Indianapolis, IN, USA.*

*^3^Stark Neurosciences Research Institute, Indiana University School of Medicine, Indianapolis, IN, USA.*

*^4^Department of Neurology, Indiana University School of Medicine, Indianapolis, IN, USA.*

*^5^Department of Psychiatry, Indiana University School of Medicine, Indianapolis, IN, USA.*

*^6^Department of Medical & Molecular Genetics, Indiana University School of Medicine, Indianapolis, IN, USA.*

*^7^Michigan Concussion Center, University of Michigan, Ann Arbor, MI, USA.*

*^8^Matthew Gfeller Center, Department of Exercise and Sport Science, University of North Carolina, Chapel Hill, NC, USA.*

*^9^Sports Medicine, University of California Los Angeles, Los Angeles, CA, USA.*

*^10^United States Air Force Academy, 2355 Faculty Drive, Suite 1N207, USAFA, CO, USA.*

*^11^Utah Valley Orthopedics and Sports Medicine, Provo, Utah; Saratoga Springs, Utah, USA.*

*^12^Department of Family Medicine, Uniformed Services University of the Health Sciences, Bethesda, MD, USA.*

Co-corresponding authors:

Kaitlyn M. Dybing: kdybing@iu.edu. 0000-0003-1790-4121

Shannon L. Risacher: srisache@iupui.edu

**Supplementary table 1** Number of participants in the sample of 931, subdivided by sport participation.

|  | **F LOC+** | **F LOC-** | **M LOC+** | **M LOC-** | **N (%)** |
| --- | --- | --- | --- | --- | --- |
| Total number of participants | 15 | 341 | 24 | 551 | 931 |
| *Sport* | | | | | |
| Baseball | - | - | 1 | 21 | 22 |
| Basketball | 1 | 35 | - | 18 | 54 |
| Beach volleyball | - | 1 | - | - | 1 |
| Boxing | - | - | - | 2 | 2 |
| Cheerleading | - | 17 | - | 4 | 21 |
| Cross country/track | - | 6 | - | 31 | 37 |
| Diving | - | 11 | - | 6 | 17 |
| Fencing | - | 2 | - | 1 | 3 |
| Field event | - | 6 | - | 2 | 8 |
| Field hockey | 1 | 16 | - | - | 17 |
| Football | - | - | 5 | 262 | 267 |
| Golf | - | 2 | - | - | 2 |
| Gymnastics | 3 | 12 | - | 5 | 20 |
| Ice hockey | - |  | - | 15 | 15 |
| Lacrosse | - | 24 | 2 | 29 | 55 |
| Rowing/crew | - | 7 | - | - | 7 |
| Rugby | - | 11 | 2 | 13 | 26 |
| Soccer | 7 | 59 | 5 | 50 | 121 |
| Softball | - | 23 | - | - | 23 |
| Sprint football | - | - | - | 1 | 1 |
| Swimming | - | 12 | - | 7 | 19 |
| Tennis | - | 5 | 1 | 4 | 10 |
| Track/field | 3 | - | - | - | 3 |
| Volleyball | - | 39 | - | 5 | 44 |
| Water polo | - | 18 | - | 8 | 26 |
| Wrestling | - | - | 1 | 22 | 23 |
| Blank/military student | - | 35 | 7 | 45 | 87 |

**Supplementary table 2** Number of participants from full GWAS sample (n = 4108) and the subsample of participants in this project (n = 931) who had missing data for 37 included alleles (excluded alleles: rs2732703 (duplicated), rs616338). In the full GWAS sample, the average number of missed SNPs for a participant was 2.44, and in the study sample the average number of missed SNPs for a participant was 2.48.

| rsID | # participants missing SNP in full GWAS (n = 4108) | # participants missing SNP in study sample (n = 931) |
| --- | --- | --- |
| rs4844610 | 43 | 9 |
| rs876461 | 104 | 21 |
| rs6733839 | 46 | 12 |
| rs10933431 | 1481 | 315 |
| rs4351014 | 390 | 88 |
| rs9275152 | 57 | 9 |
| rs143332484 | 0 | 0 |
| rs75932628 | 2 | 1 |
| rs9381040 | 16 | 3 |
| rs9381564 | 340 | 85 |
| rs1859788 | 1073 | 252 |
| rs56402156 | 0 | 0 |
| rs73223431 | 50 | 12 |
| rs9331896 | 26 | 7 |
| rs34674752 | 320 | 79 |
| rs34173062 | 890 | 210 |
| rs7920721 | 120 | 22 |
| rs3740688 | 122 | 31 |
| rs1582763 | 183 | 48 |
| rs3851179 | 6 | 2 |
| rs11218343 | 12 | 5 |
| rs17125924 | 109 | 21 |
| rs11623019 | 200 | 48 |
| rs593742 | 720 | 160 |
| rs117618017 | 798 | 210 |
| rs7185636 | 993 | 205 |
| rs4985556 | 484 | 119 |
| rs12444183 | 94 | 18 |
| rs3935877 | 270 | 57 |
| rs72824905 | 40 | 12 |
| rs72835061 | 84 | 19 |
| rs75511804 | 0 | 0 |
| rs4311 | 18 | 2 |
| rs3752231 | 286 | 76 |
| rs12459419 | 48 | 8 |
| rs6024870 | 135 | 34 |
| rs2154481 | 477 | 113 |

**Supplementary table 3** Summary of outcomes of linear regression analyses with covariates (PRS = polygenic risk score; RTP = return to play; BESS = balance error scoring system; SAC = standardized assessment of concussion; SCATSEV = symptom severity score on the sport concussion assessment tool (SCAT); SCATSYMP = total number of symptoms score on the SCAT; LOC = loss of consciousness; Mil = participant of military origin; Civ = participant of civilian origin, EAS = East Asians, EUR = Europeans, AFR = Africans, AMR = Admixed Americans, SAS = South Asians).

| Analysis | Estimate (β) | Standard error (SE) | T value | P | R^2^ | Covariate F value (p) |
| --- | --- | --- | --- | --- | --- | --- |
| PRS & days to normal RTP | 0.349 | 0.180 | 1.935 | 0.054 | Mult. 0.062  Adj. 0.050 | Ancestry 1.564 (0.182)  Sex 1.327 (0.250)  LOC 2.001 (0.136)  Mil vs. Civ 28.801 (**<0.001**) (mil>civ; estimate = 2.210, SE = 0.413) |
| PRS & days to long RTP | 0.013 | 2.790 | 0.005 | 0.996 | Mult. 0.024  Adj. -0.012 | Ancestry 1.444 (0.231)  Sex 0.011 (0.916)  LOC 0.077 (0.926)  Mil vs. Civ 0.772 (0.381) |
| PRS & days to asymptomatic | -0.337 | 0.413 | -0.816 | 0.415 | Mult. 0.053  Adj. 0.043 | Ancestry 2.684 (**0.030**) (EAS>EUR, estimate = 5.297, SE = 1.76)  Sex 10.044 (**0.002**) (female>male, estimate = 3.360, SE = 0.872)  LOC 0.1065 (0.899)  Mil vs. Civ 25.9778 (**<0.001**) (mil>civ; estimate = 4.750, SE = 0.932) |
| PRS & BESS total score | 0.124 | 0.251 | 0.495 | 0.621 | Mult. 0.047  Adj. 0.037 | Ancestry 0.751 (0.557)  Sex 0.905 (0.342)  LOC 13.170 (**<0.001**) (no LOC>unknown LOC status; estimate = 3.460, SE = 0.752)  Mil vs. Civ 12.607 (**<0.001**) (mil>civ; estimate = 2.00, SE = 0.563) |
| PRS & SAC total score | 0.001 | 0.080 | 0.018 | 0.986 | Mult. 0.040  Adj. 0.030 | Ancestry 1.413 (0.540)  Sex 0.148 (0.228)  LOC 12.288 (**<0.001**) (LOC & no LOC<unknown; estimates = 1.805 & 1.158, SE = 0.444 & 0.242)  Mil vs. Civ 7.011 (**0.008**) (mil>civ; estimate = 0.474, SE = 0.179) |
| PRS & SCATSYMP | -0.137 | 0.273 | -0.503 | 0.615 | Mult. 0.019  Adj. 0.002 | Ancestry 0.386 (0.763)  Sex 2.494 (0.115)  LOC 2.351 (0.096)  Mil vs. Civ 0.057 (0.812) |
| PRS & SCATSEV | -0.202 | 0.895 | -0.226 | 0.821 | Mult. 0.020  Adj. 0.003 | Ancestry 0.874 (0.454)  Sex 2.600 (0.108)  LOC 1.776 (0.171)  Mil vs. Civ 0.357 (0.551) |

**Supplementary table 4** Results from ANOVAs of outcomes by apolipoprotein E (*APOE*) genotype (ε3/ε3 vs ε2 carriers vs ε4 carriers), with covariates of sex (male vs. female), loss of consciousness (LOC) status, participant type (military or civilian origin), and genetic ancestry (BESS = balance error scoring system; RTP = return to play; SAC = standardized assessment of concussion; SCATSEV = symptom severity score on the sport concussion assessment tool (SCAT); SCATSYMP = total number of symptoms score on the SCAT, Mil = participant of military origin; Civ = participant of civilian origin, EAS = East Asians, EUR = Europeans, AFR = Africans, AMR = Admixed Americans, SAS = South Asians)

| Analysis | n participants | F value (p) | Covariate F value (p) |
| --- | --- | --- | --- |
| Normal RTP | 417 ε3/ε3  77 ε2 carriers  183 ε4 carriers | 1.099 (0.334) | Ancestry 0.588 (0.443)  Sex 7.989 (**0.005**)  LOC 3.270 (0.071)  Mil vs. Civ 27.242 (**<0.001**) |
| Long RTP | 131 ε3/ε3  32 ε2 carriers  60 ε4 carriers | 0.107 (0.899) | Ancestry 2.226 (0.137)  Sex 0.057 (0.811)  LOC 0.084 (0.773)  Mil vs. Civ 0.591 (0.443) |
| Time to asymptomatic | 508 ε3/ε3  98 ε2 carriers  221 ε4 carriers | 0.186 (0.831) | Ancestry 5.972 (**0.015**)  Sex 14.622 (**<0.001**)  LOC 0.047 (0.828)  Mil vs. Civ 23.198 (**<0.001**) |
| BESS total score | 528 ε3/ε3  101 ε2 carriers  230 ε4 carriers | 0.089 (0.915) | Ancestry 1.448 (0.229)  Sex 0.970 (0.325)  LOC 7.008 (**0.008**)  Mil vs. Civ 14.529 (**<0.001**) |
| SAC total score | 541 ε3/ε3  109 ε2 carriers  238 ε4 carriers | 0.104 (0.901) | Ancestry 0.365 (0.546)  Sex 3.091 (0.079)  LOC 23.366 (**<0.001**)  Mil vs. Civ 6.555 (**0.011**) |
| SCATSYMP | 280 ε3/ε3  55 ε2 carriers  123 ε4 carriers | 0.396 (0.673) | Ancestry 0.189 (0.664)  Sex 2.024 (0.155)  LOC 4.255 (**0.040**)  Mil vs. Civ 0.018 (0.895) |
| SCATSEV | 280 ε3/ε3  55 ε2 carriers  123 ε4 carriers | 1.336 (0.264) | Ancestry 0.046 (0.830)  Sex 1.336 (0.248)  LOC 2.058 (0.152)  Mil vs. Civ 0.584 (0.445) |

**Supplementary fig. 1** Sensitivity analysis of missing SNP data handling methods. In (a) and (b), linear regressions to test the relationship between RTP and AD PRS were performed on the full 931 participants using two methods for handling missing SNP data: the ignoring method (PRS value of missing SNP is replaced with 0) (a) and the mean substitution method (PRS value of missing SNP is replaced with the mean score for that SNP from all other participants) (b). For the ignoring method, there was no significant relationship (*p* = 0.8152). The relationship approached significance using the mean substitution method (*p* = 0.05314). These tests were repeated using a subsample of participants with outliers removed by R (c and d). There were no significant relationships using the ignoring method (*p* = 0.8605) (c) or the mean substitution method (*p* = 0.7352) (d).


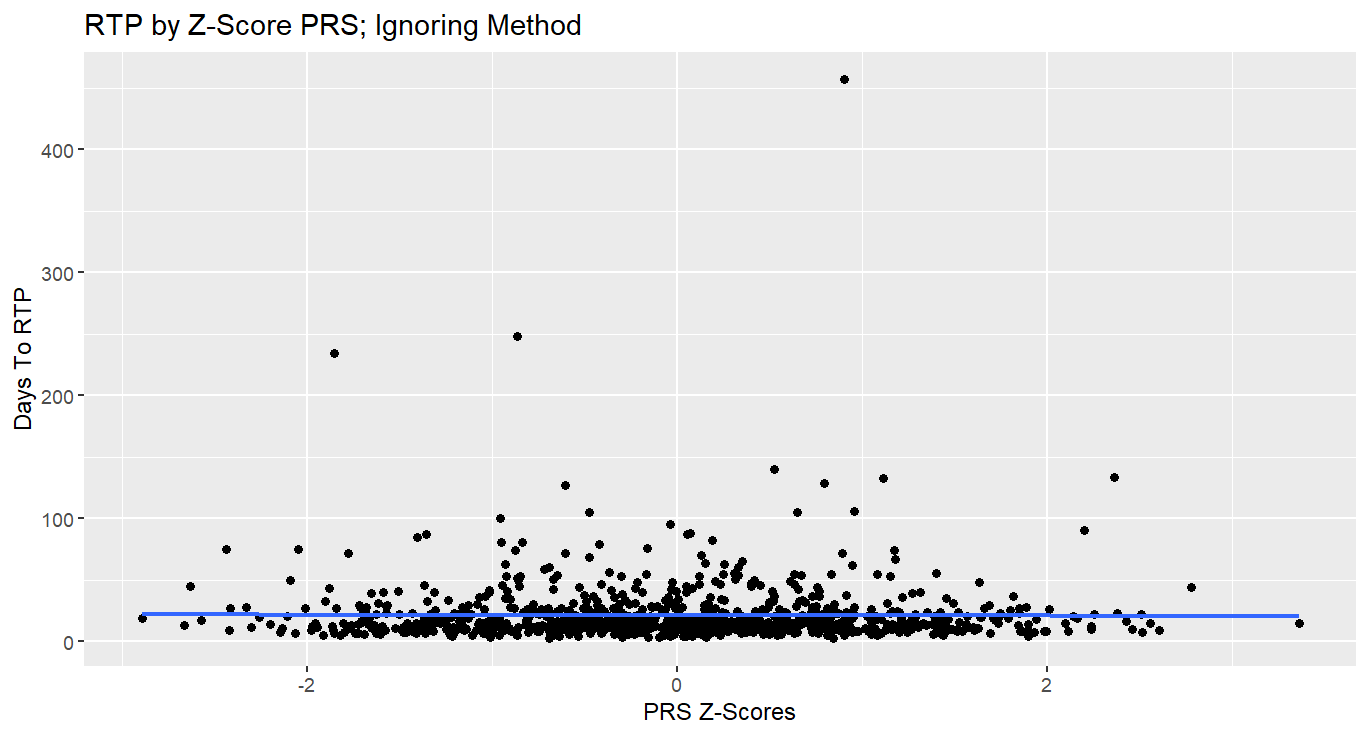
a

b


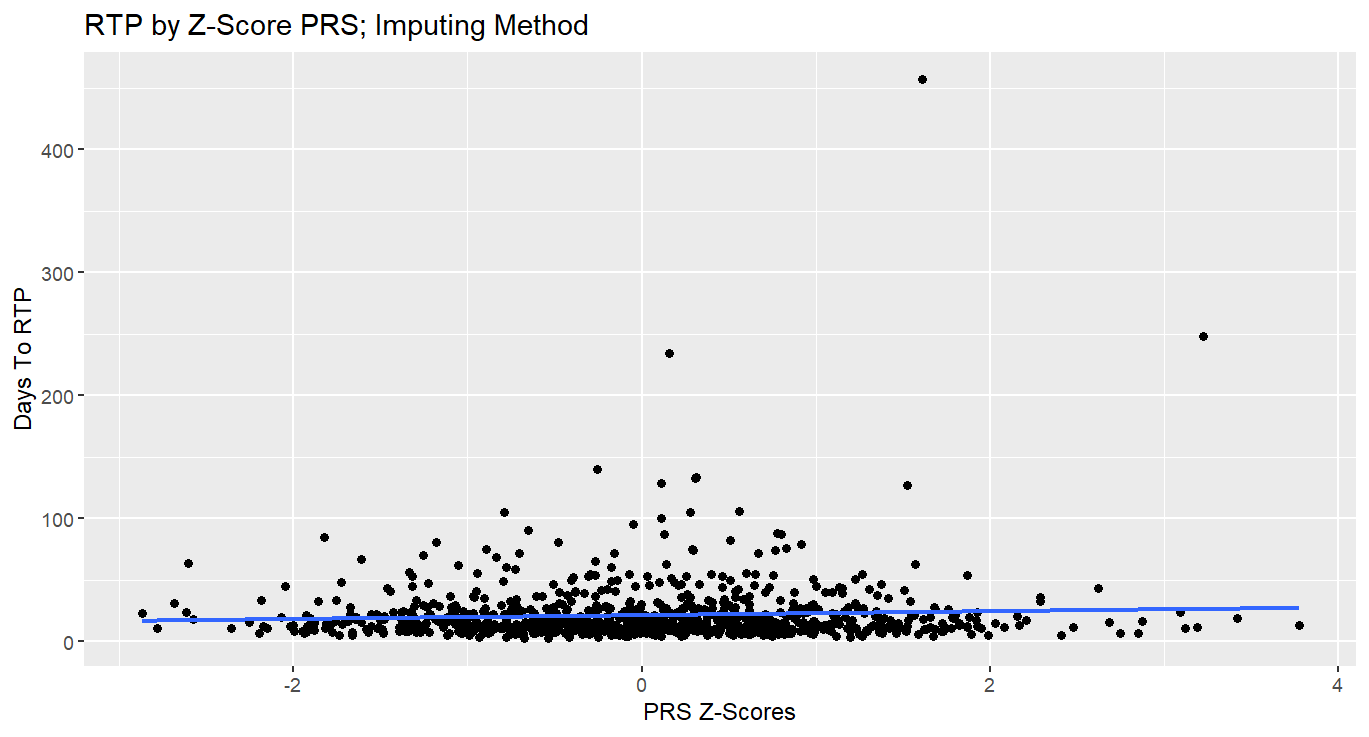


c


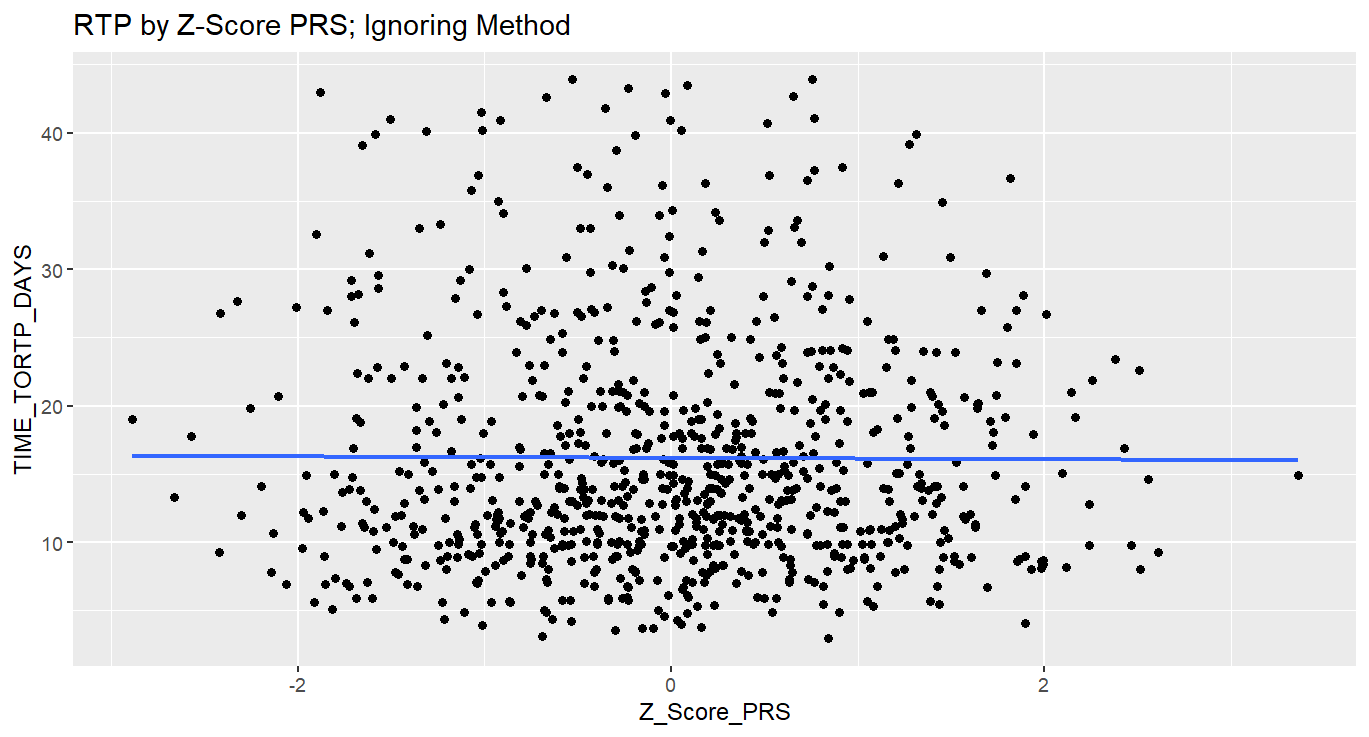


d


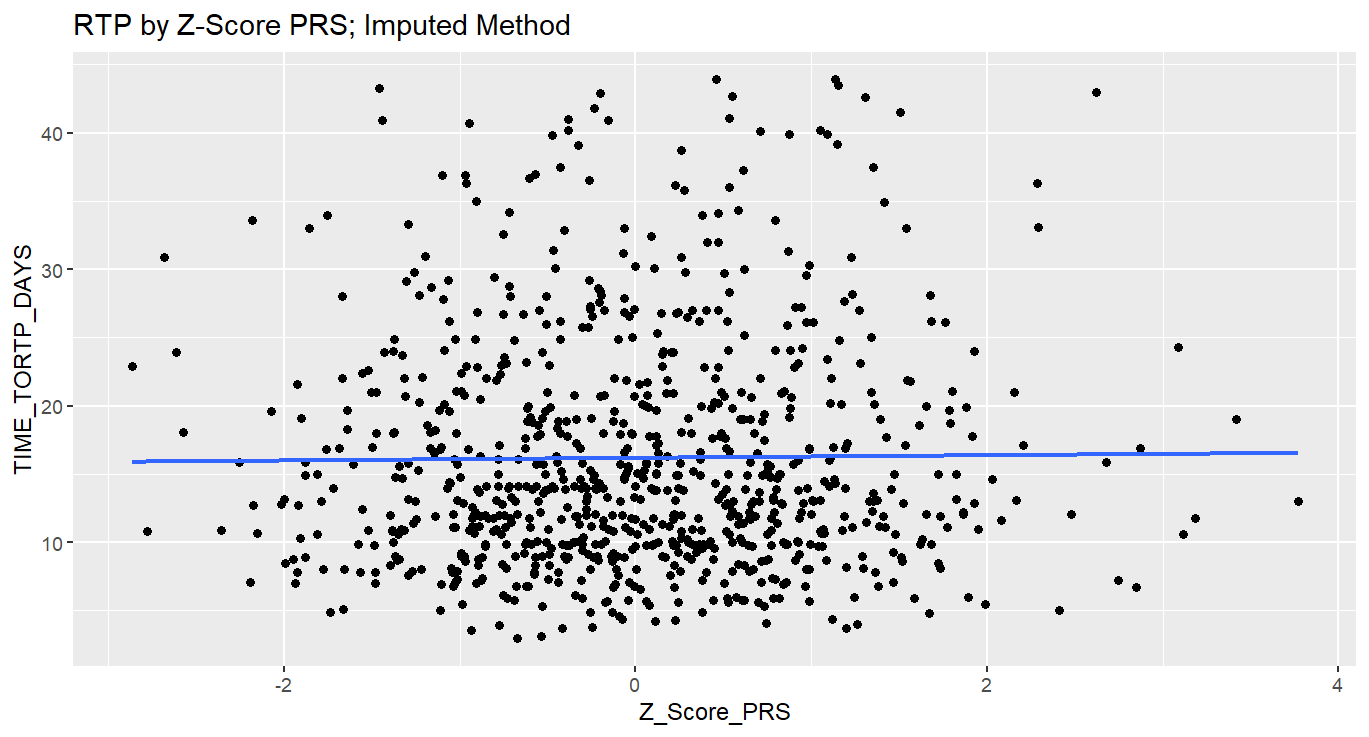


**Supplementary fig. 2** AD PRS & normal RTP interval in F LOC+ (a), F LOC- (b), M LOC+ (c), and M LOC- (d) participant subgroups.


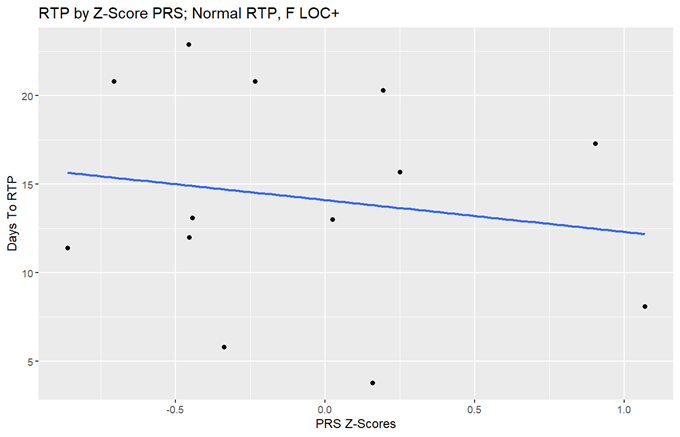
a

b


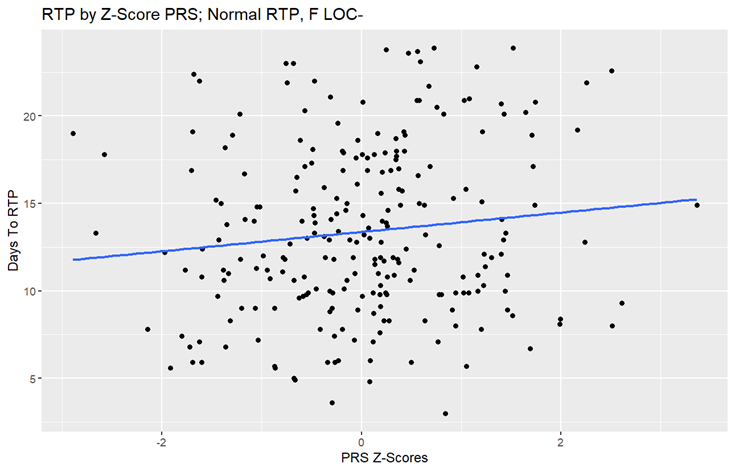


c


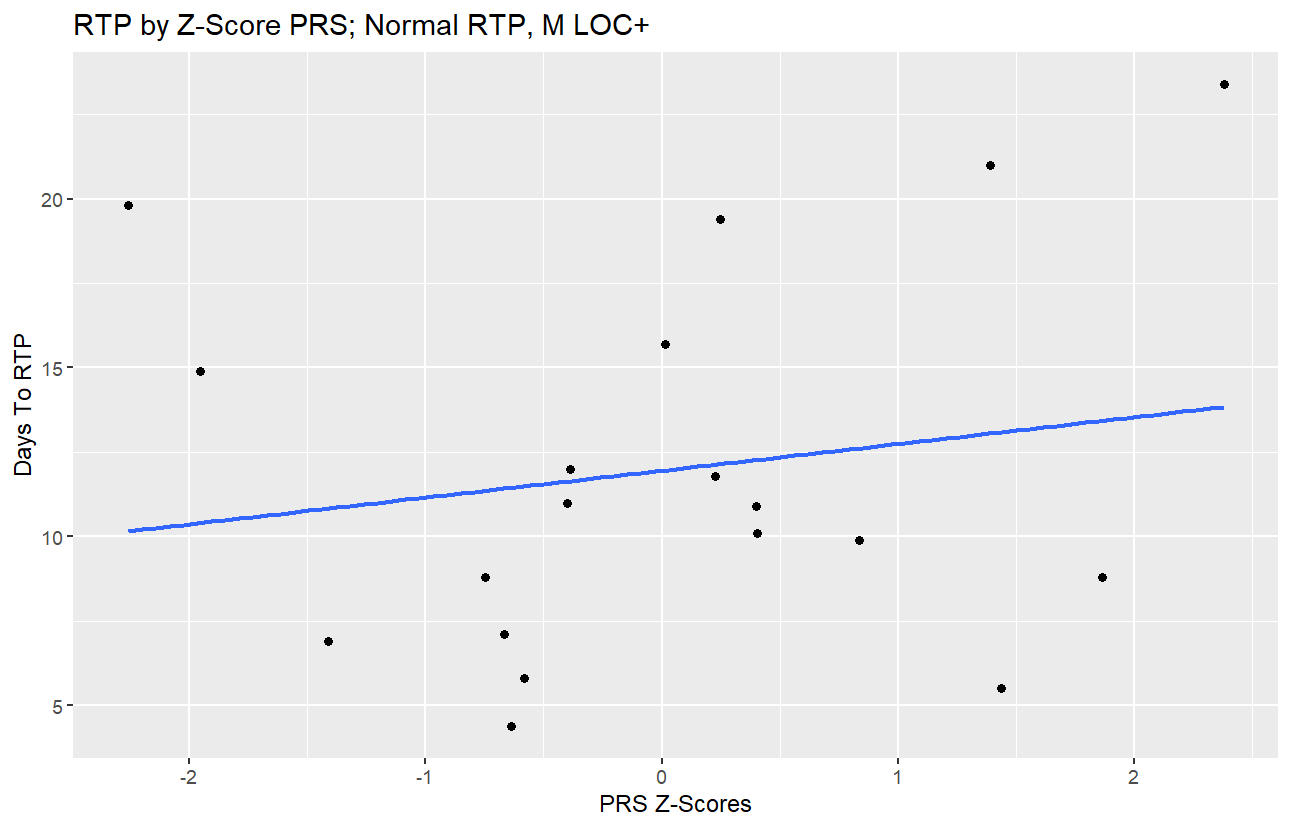


d


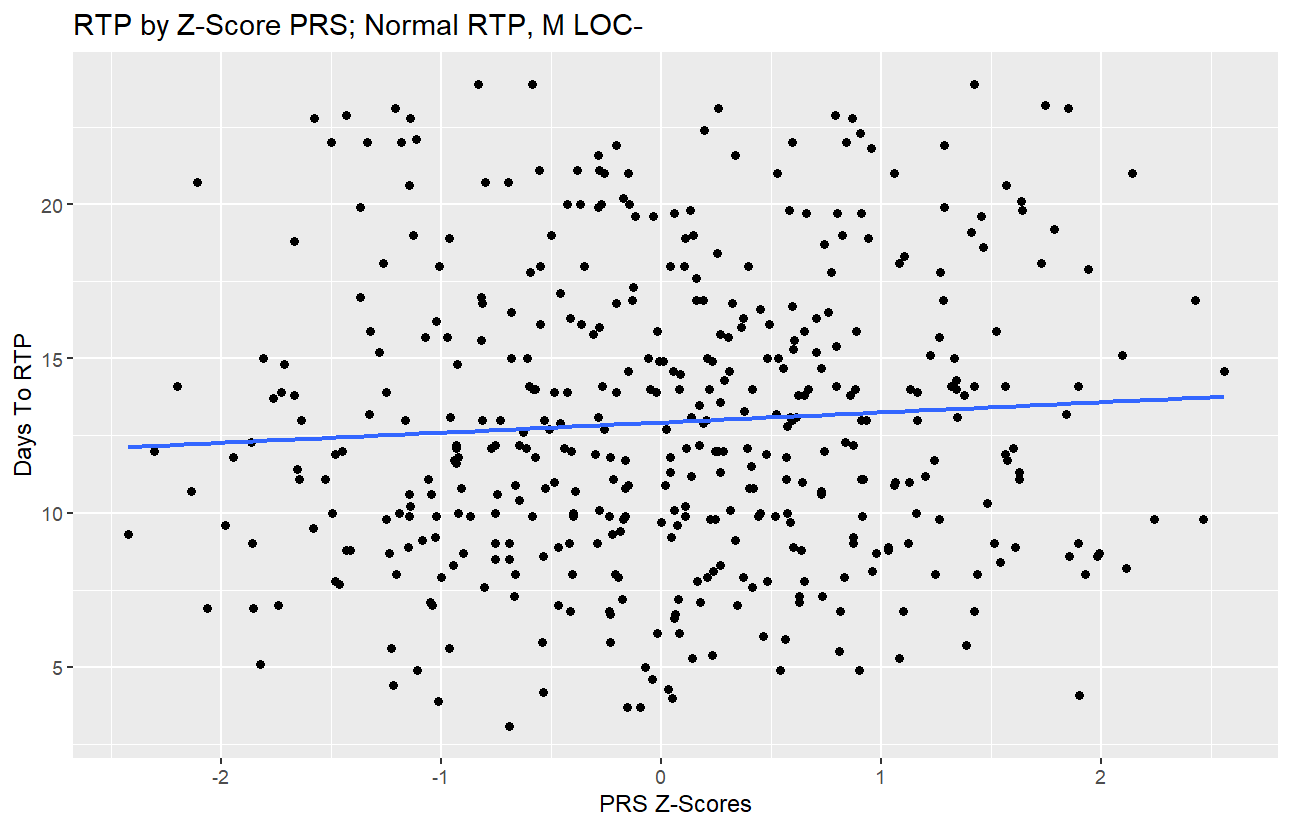


**Supplementary fig. 3** AD PRS & total scores on BESS (*p* = 0.58) (a), total scores on SAC (*p* = 0.937) (b), SCAT symptom severity scores (SCATSEV; *p* = 0.746) (c), and SCAT total number of symptom scores (SCATSYMP; *p* = 0.969) (d) in normal RTP (<24 days) data subset.

**
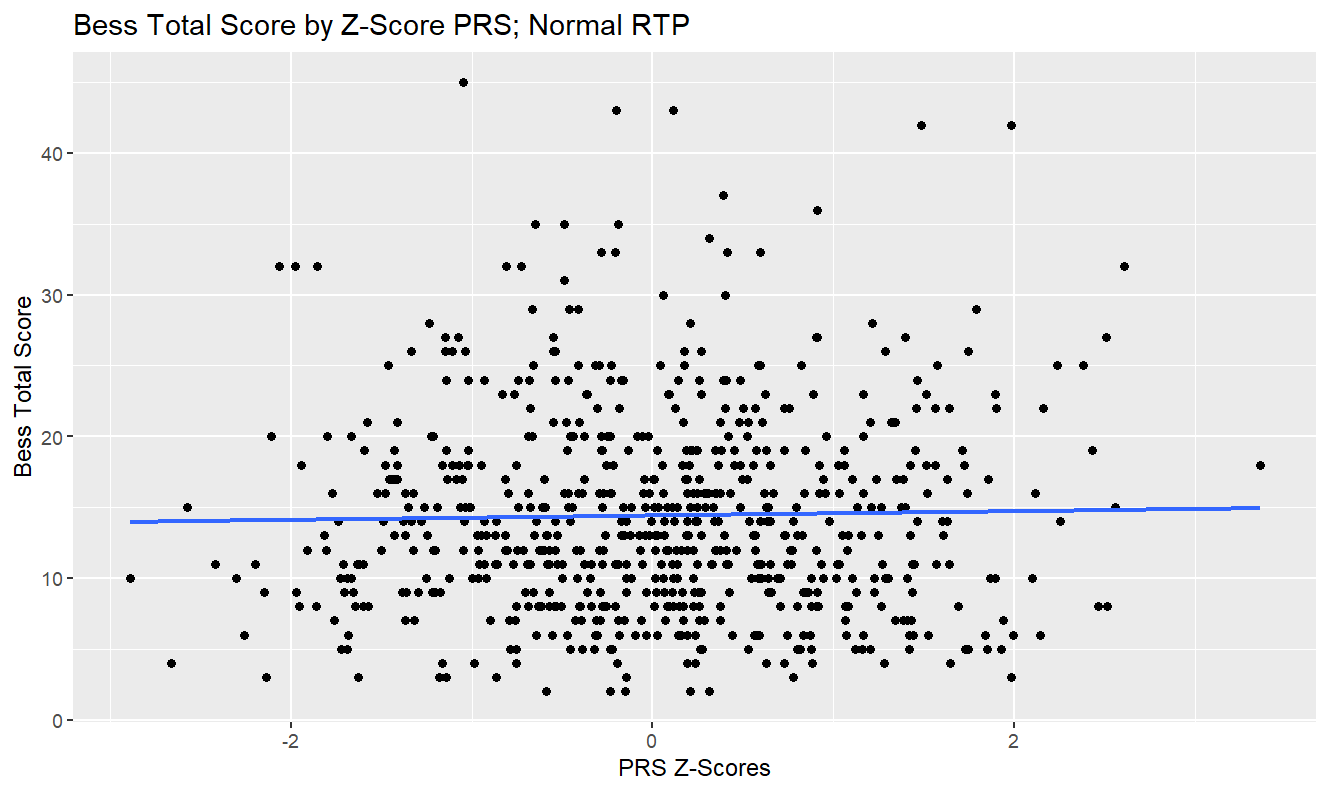
**a


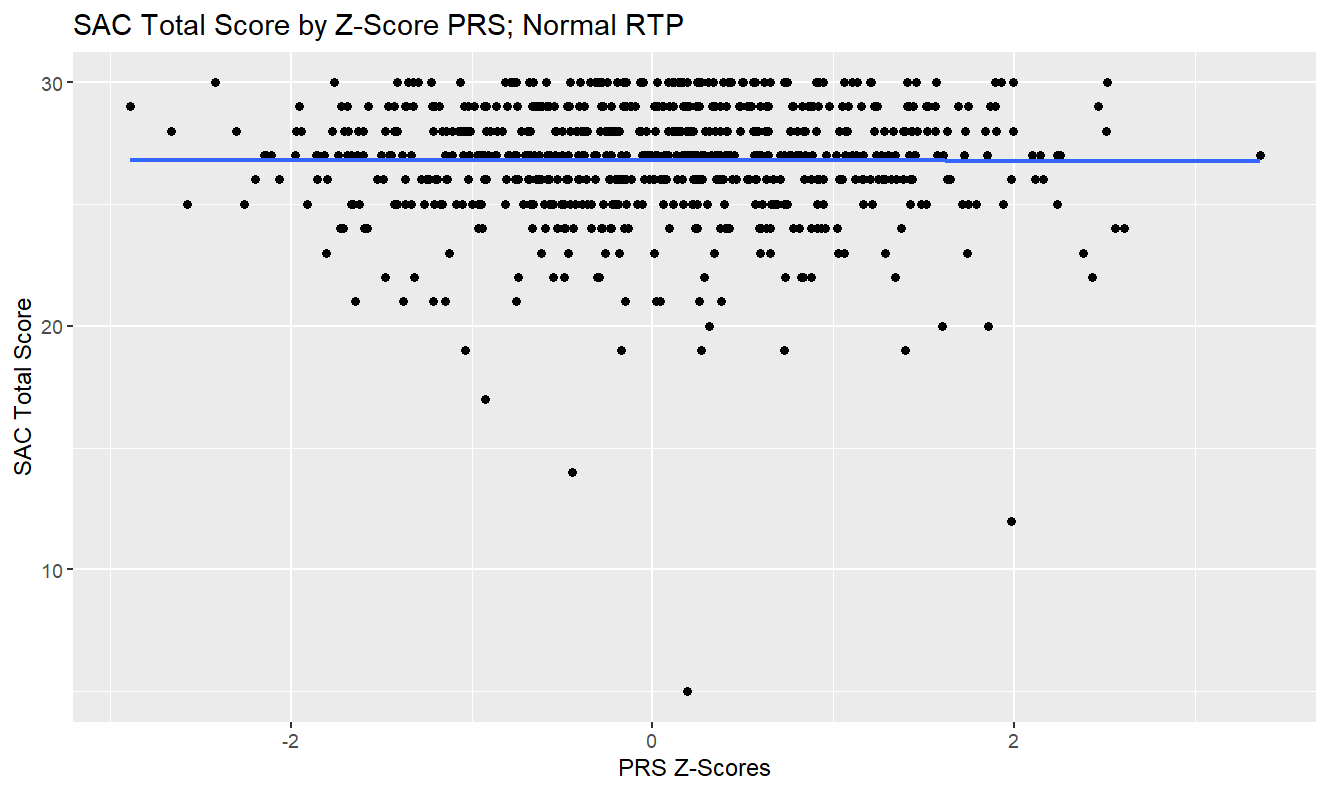
b

c

**
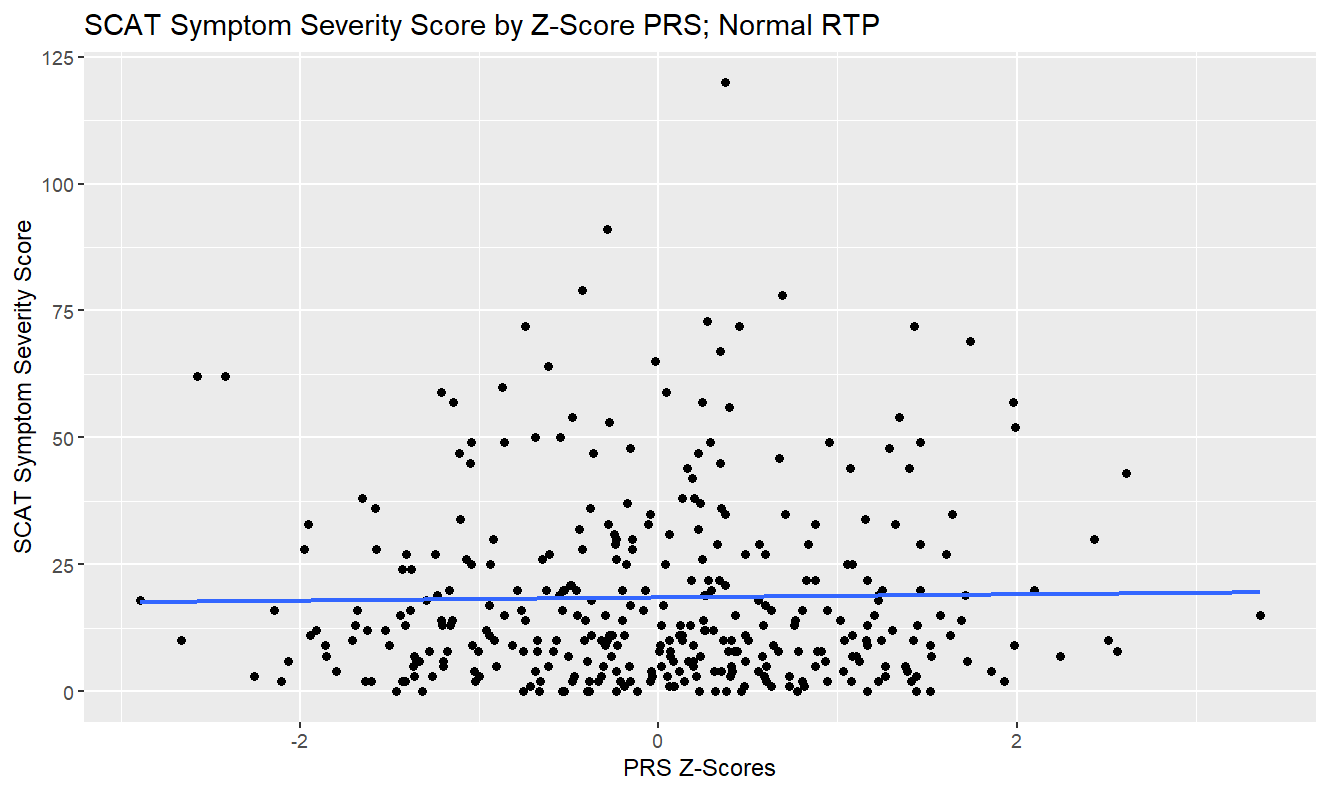
**

d

**
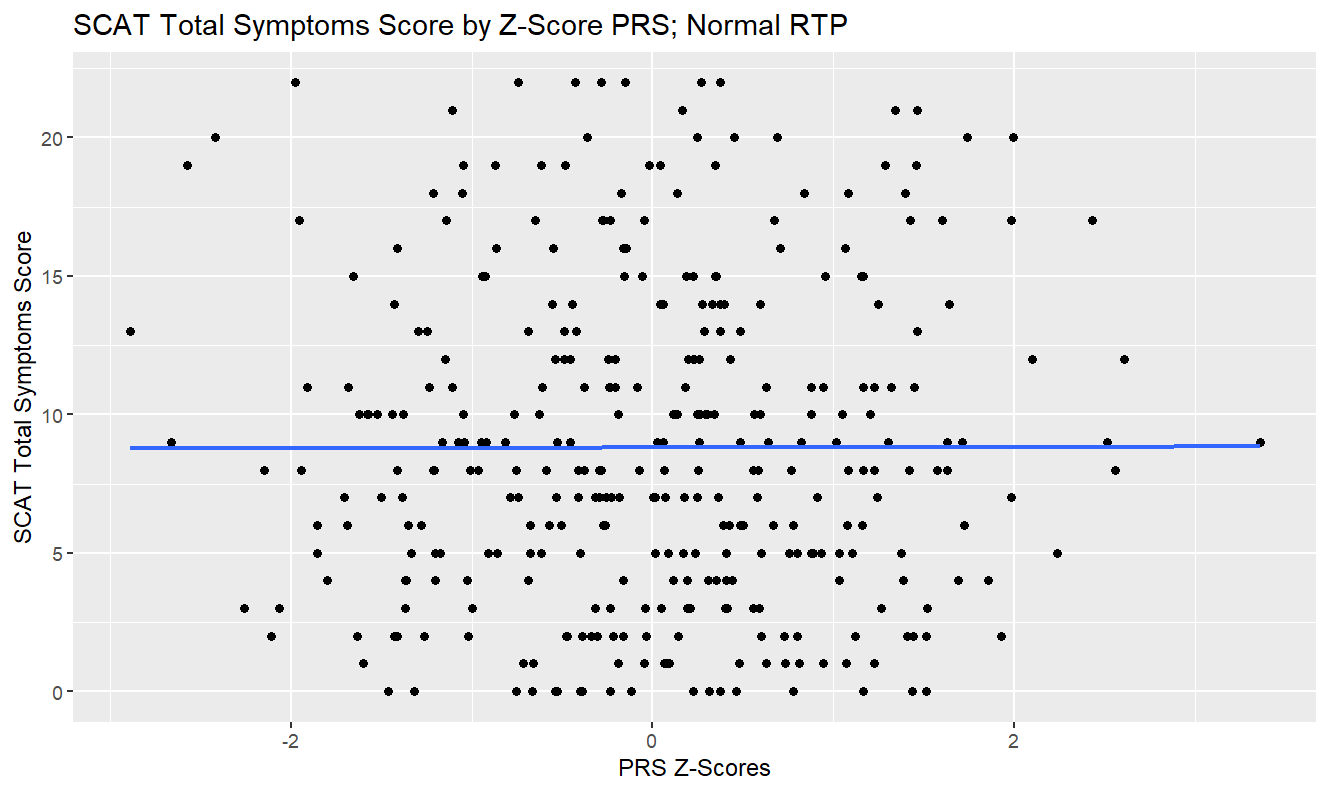
**

**Supplementary fig. 4** AD PRS & total scores on BESS (*p* = 0.645) (a), total scores on SAC (*p* = 0.117) (b), SCAT symptom severity scores (SCATSEV; *p* = 0.465) (c), and SCAT total number of symptom scores (SCATSYMP; *p* = 0.578) (d) in long RTP (>24 days) data subset.

a


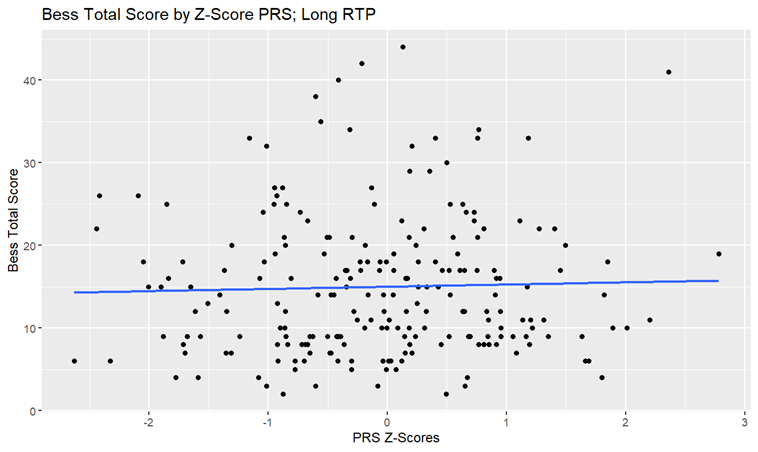


**
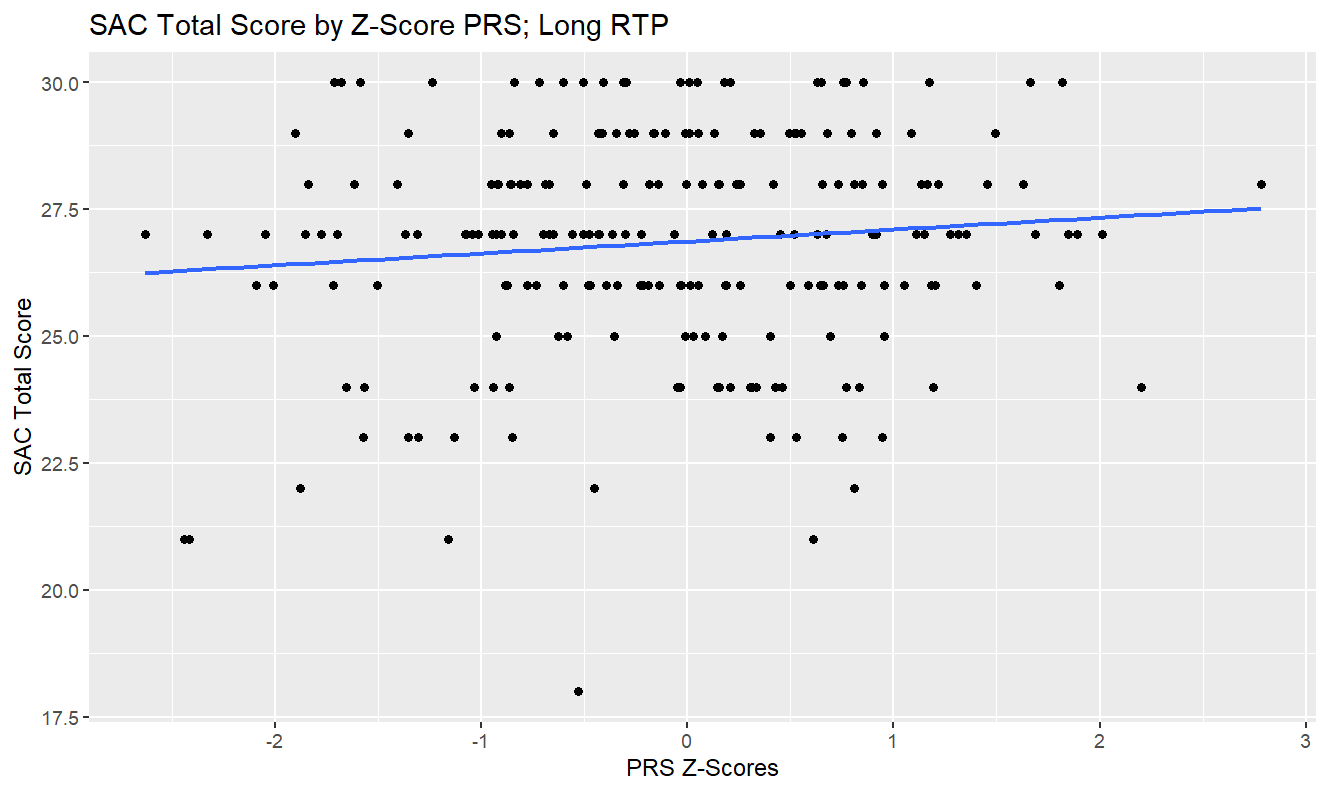
**b

c

**
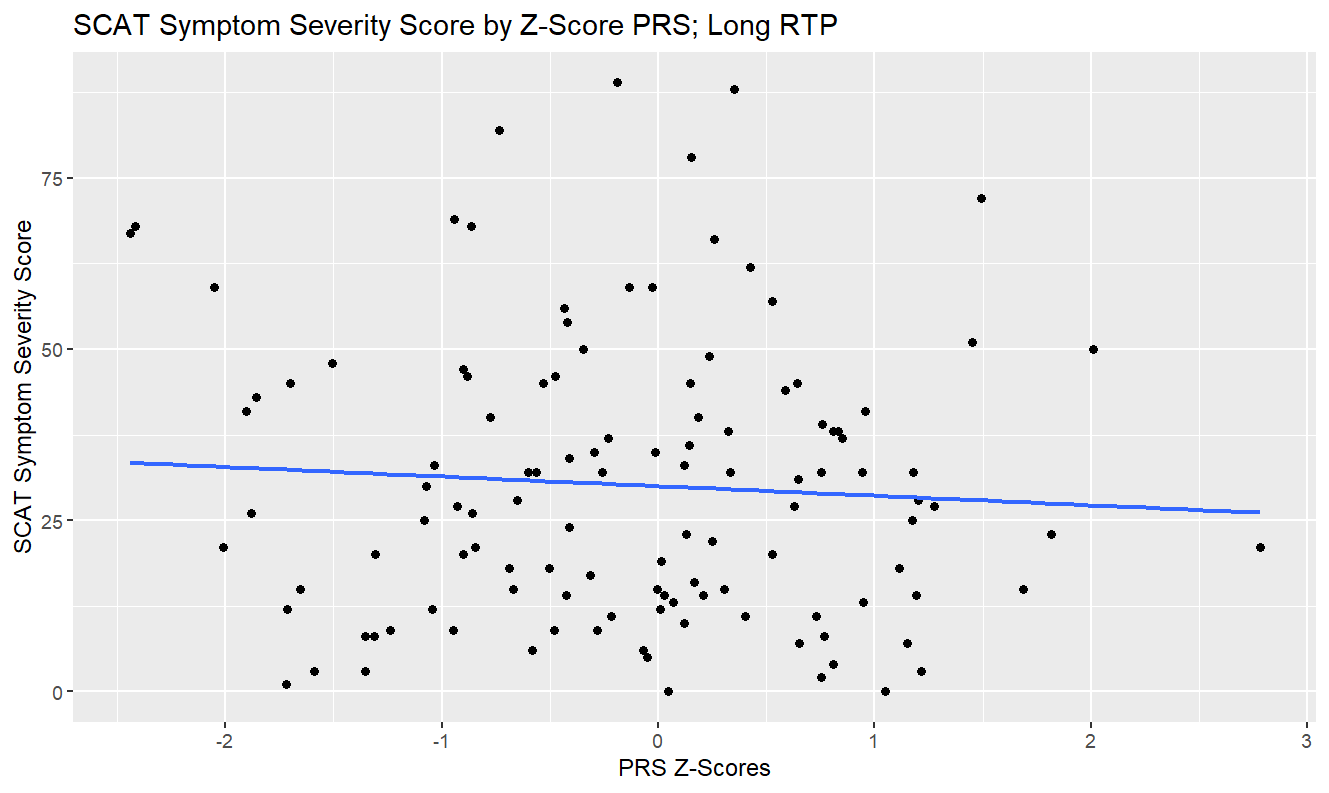
**

**
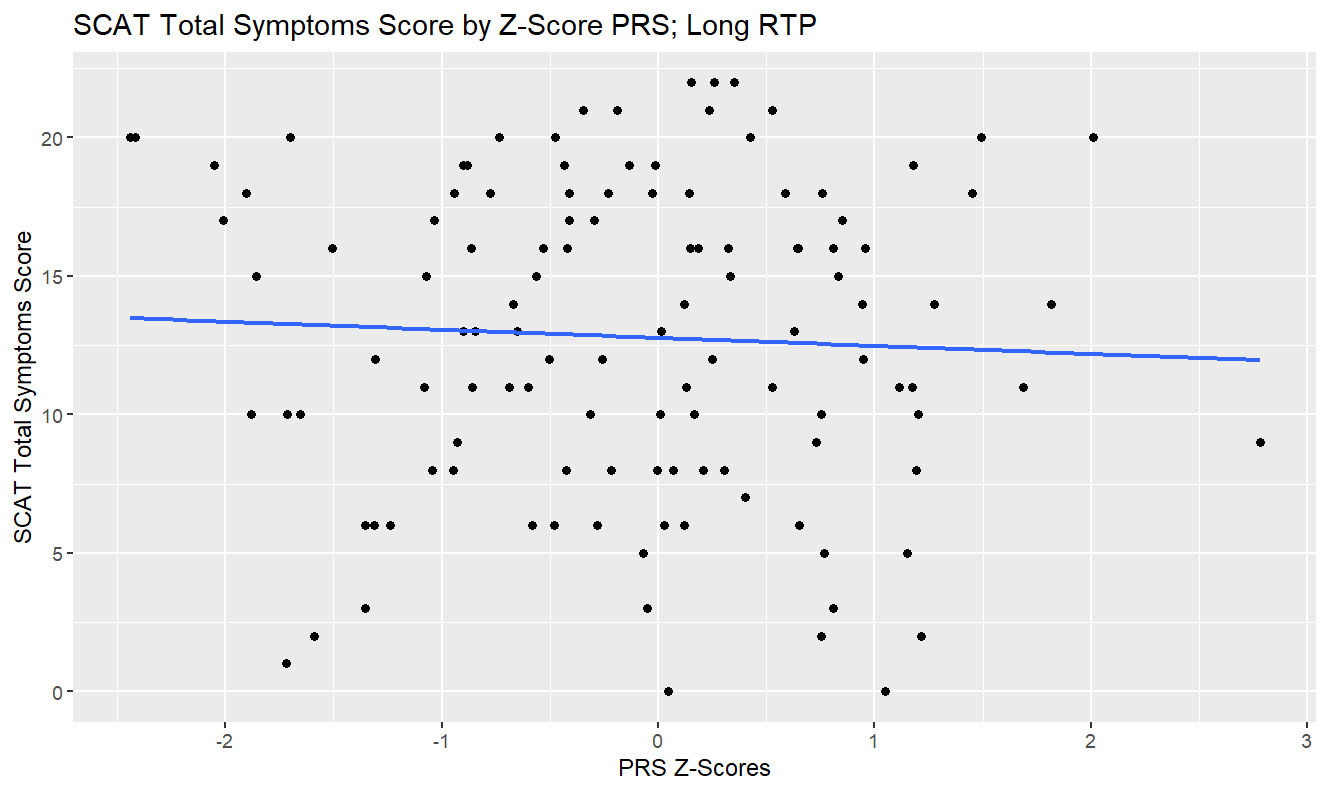
**d

**Supplementary fig. 5** AD PRS and total scores on BESS (*p* = 0.163) (a), days to normal RTP (*p* = 0.221) (b), days to long RTP (*p* = 0.446) (c), total scores on SAC (*p* = 0.715) (d), and SCAT symptom severity scores (SCATSEV; *p* = 0.144) (e) in participants of African genetic ancestry.

**
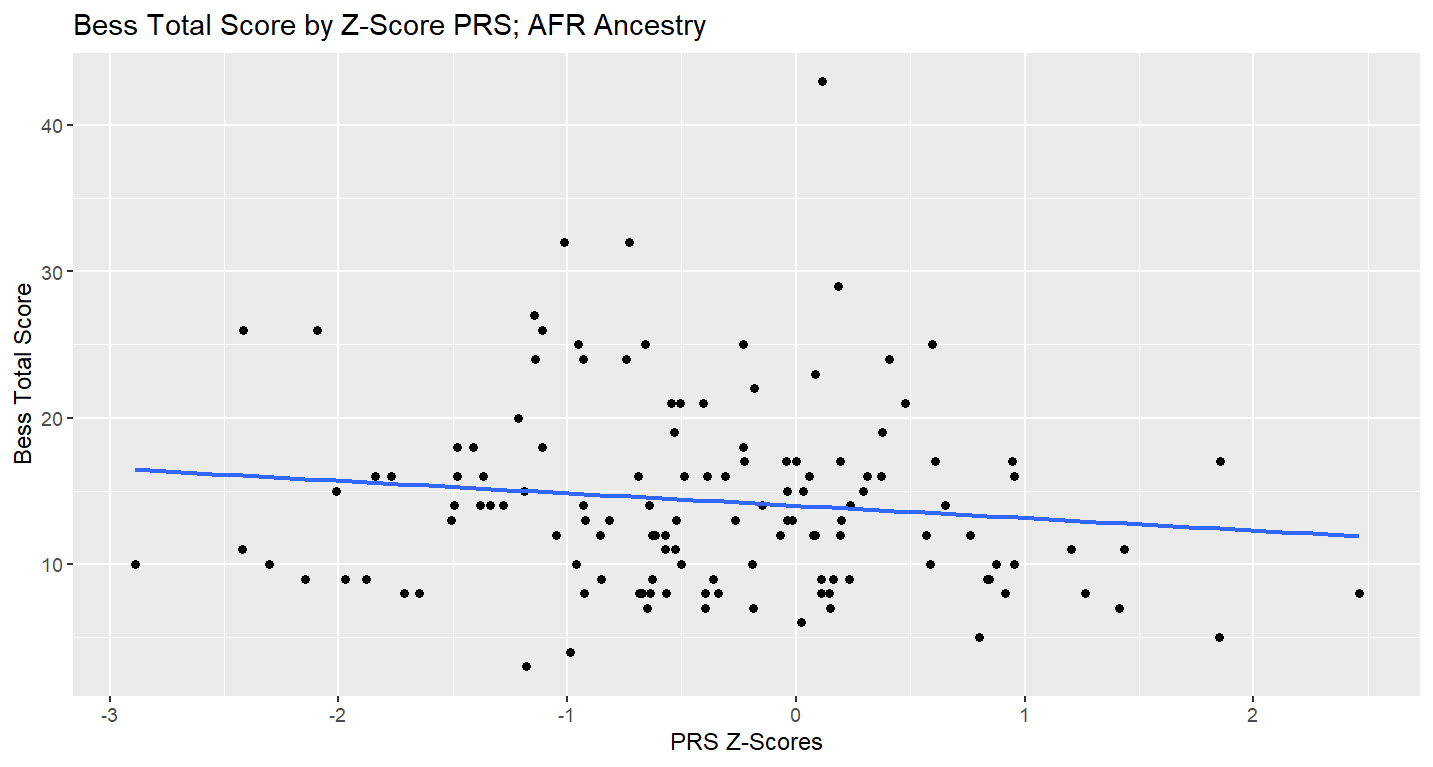
**a

b

***
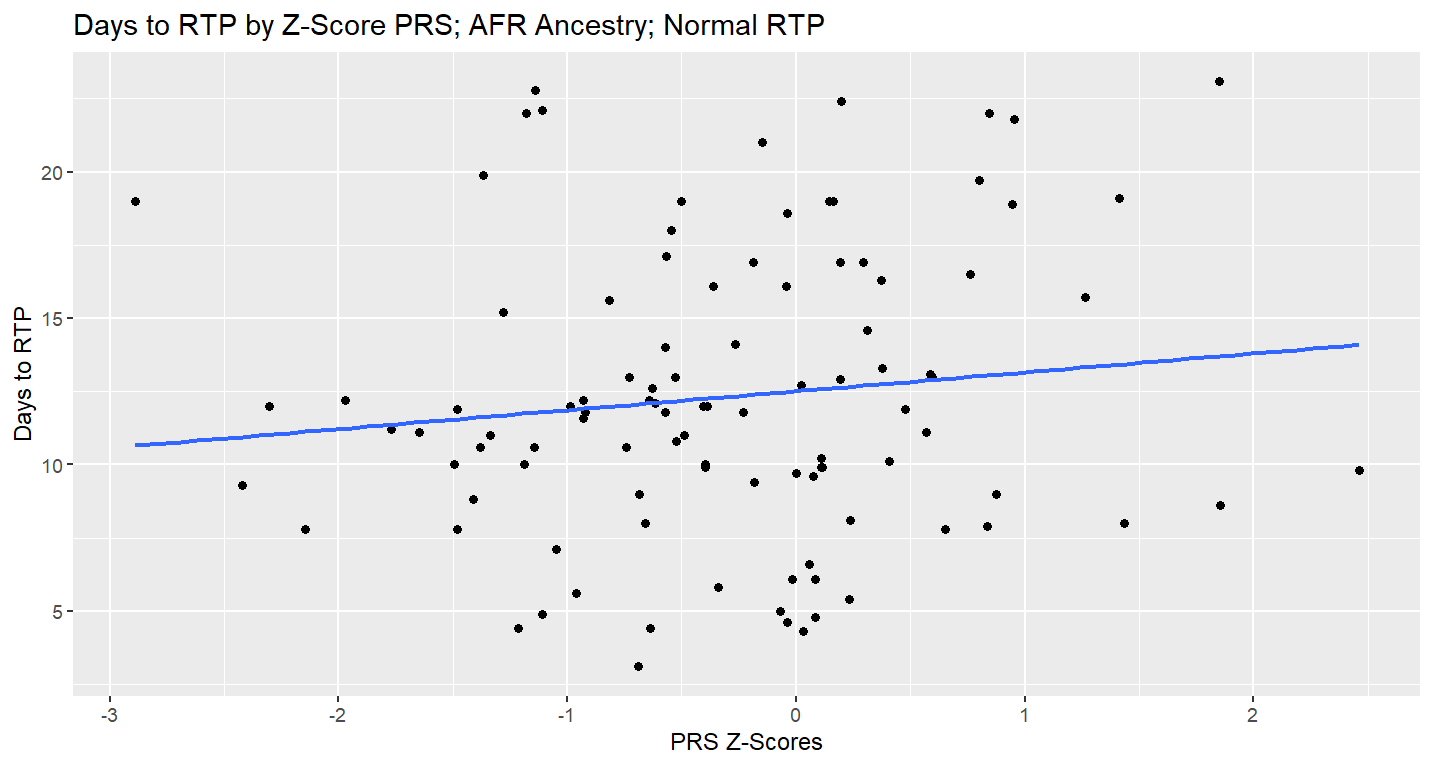
***

***
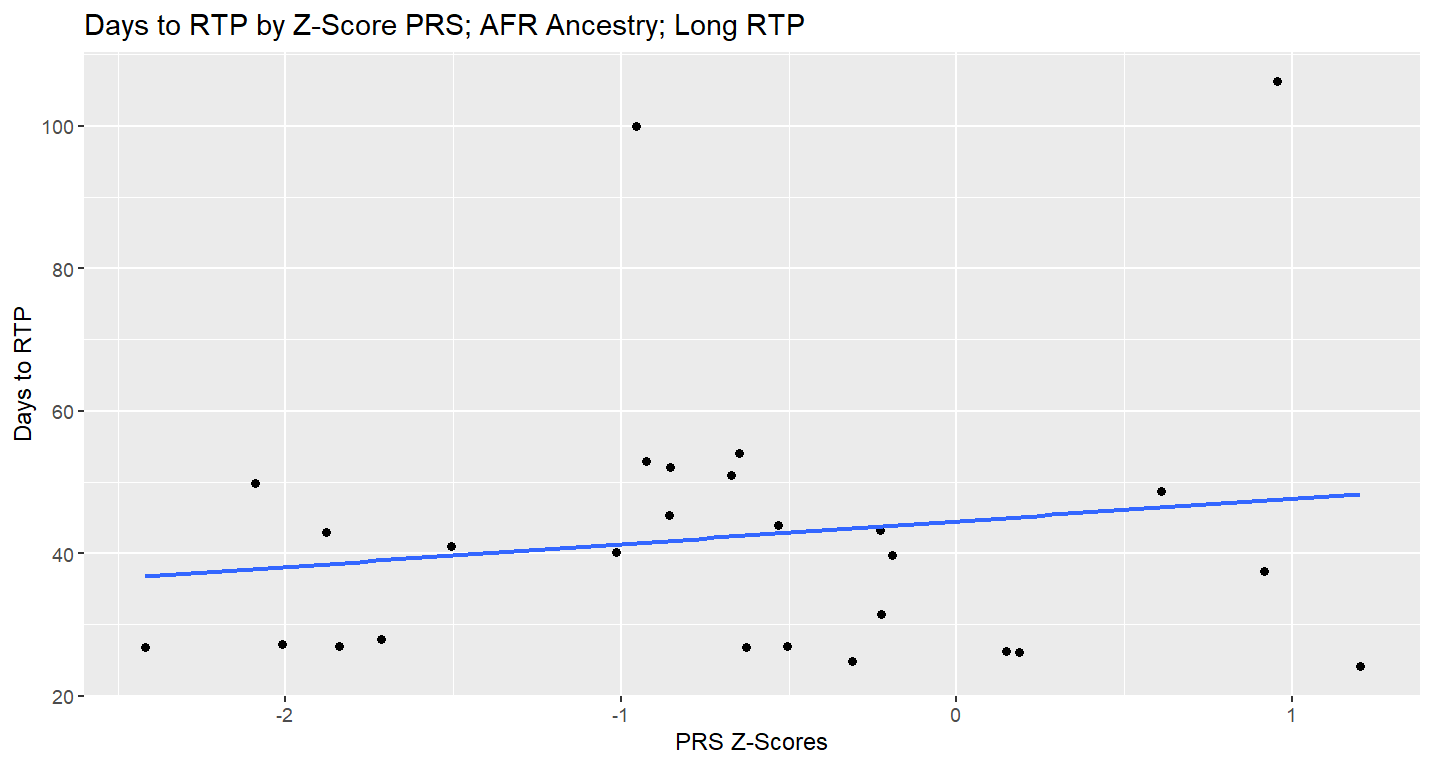
***c

**
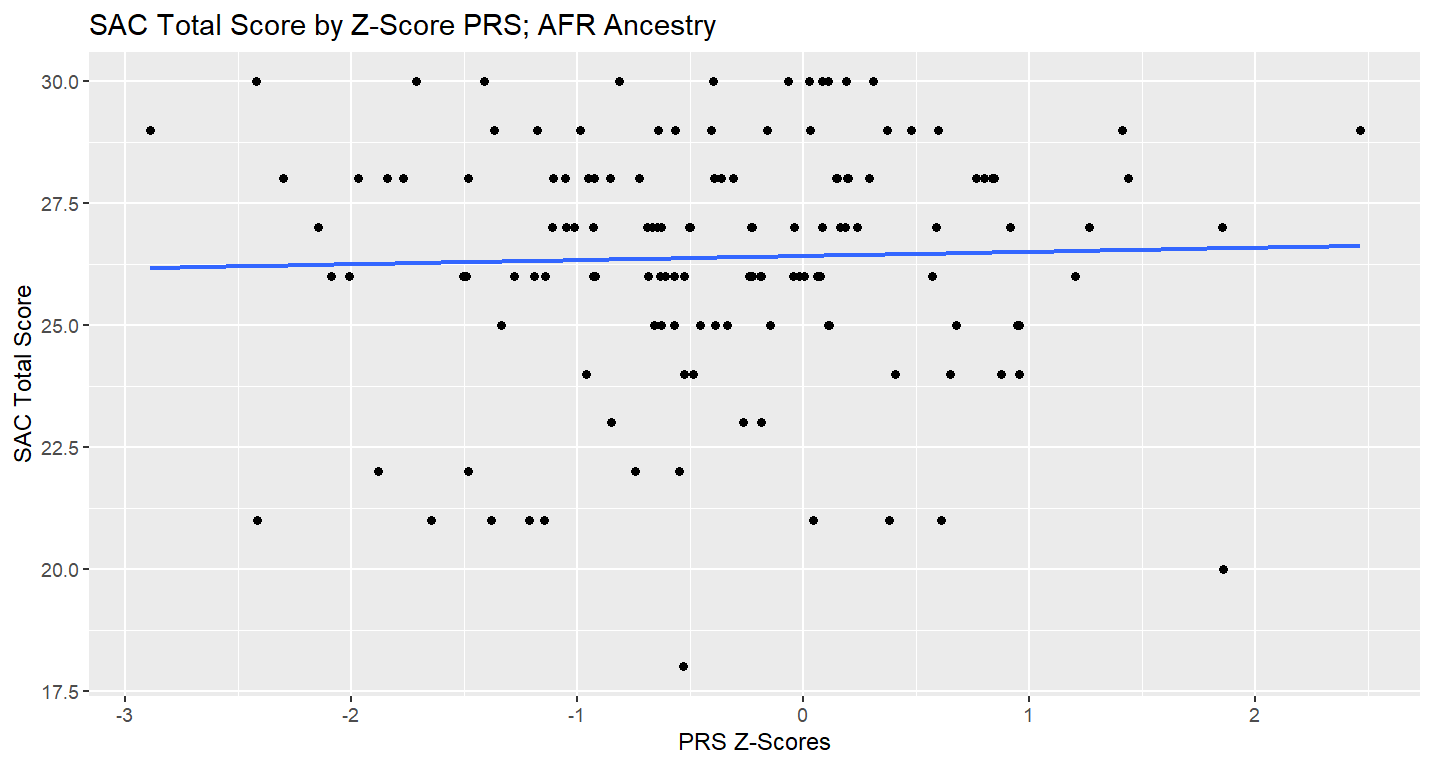
**d

**
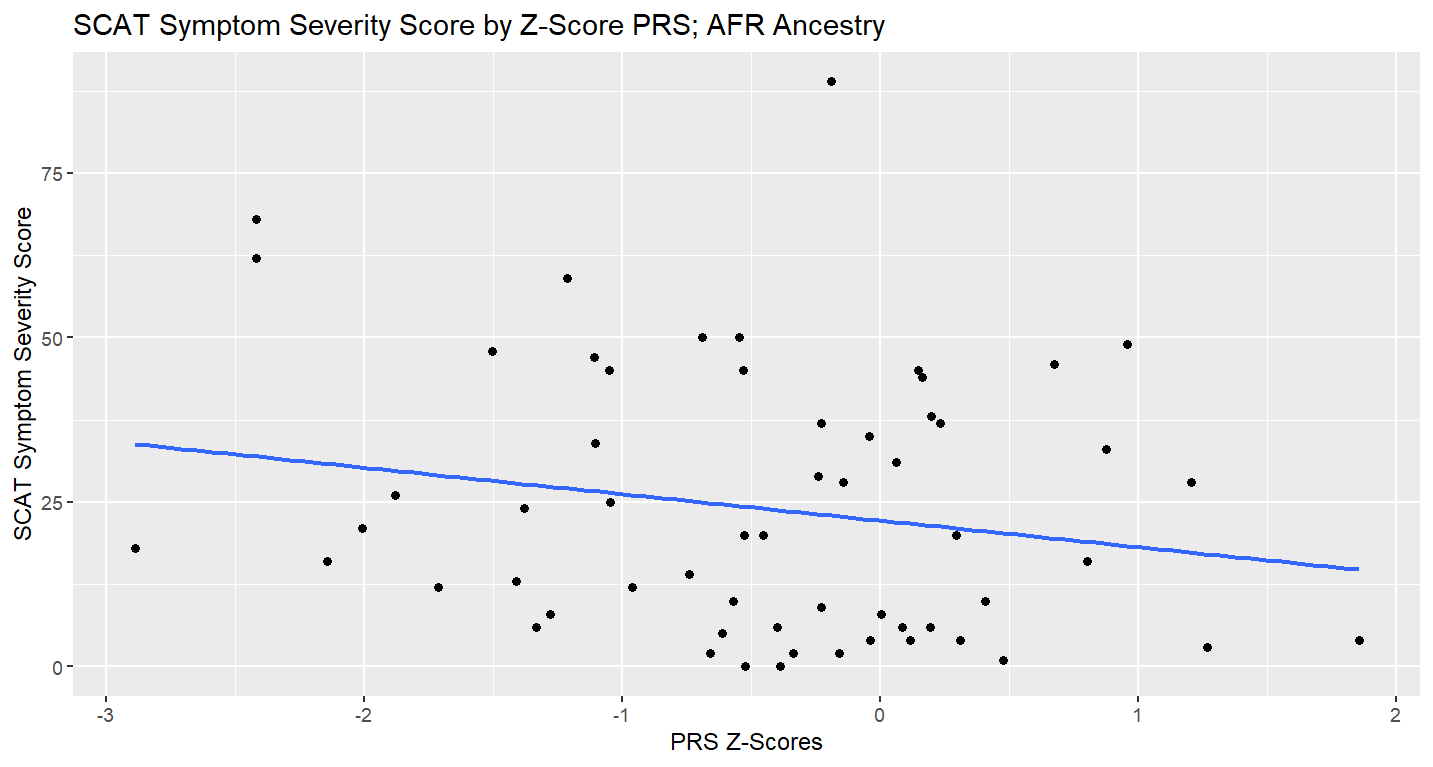
**e
